# Structure Based *In Silico* Analysis of Quinolone Resistance in Clinical Isolates of *Salmonella* Typhi from India

**DOI:** 10.1371/journal.pone.0126560

**Published:** 2015-05-11

**Authors:** Manoj Kumar, Sushila Dahiya, Priyanka Sharma, Sujata Sharma, Tej P. Singh, Arti Kapil, Punit Kaur

**Affiliations:** 1 Department of Biophysics, All India Institute of Medical Sciences, New Delhi, India; 2 Department of Microbiology, All India Institute of Medical Sciences, New Delhi, India; Second University of Naples, ITALY

## Abstract

Enteric fever is a major cause of morbidity in several parts of the Indian subcontinent. The treatment for typhoid fever majorly includes the fluoroquinolone group of antibiotics. Excessive and indiscriminate use of these antibiotics has led to development of acquired resistance in the causative organism *Salmonella* Typhi. The resistance towards fluoroquinolones is associated with mutations in the target gene of DNA Gyrase. We have estimated the Minimum Inhibitory Concentration (MIC) of commonly used fluoroquinolone representatives from three generations, ciprofloxacin, ofloxacin, levofloxacin and moxifloxacin, for 100 clinical isolates of *Salmonella* Typhi from patients in the Indian subcontinent. The MICs have been found to be in the range of 0.032 to 8 μg/ml. The gene encoding DNA Gyrase was subsequently sequenced and point mutations were observed in DNA Gyrase in the quinolone resistance determining region comprising Ser83Phe/Tyr and Asp87Tyr/Gly. The binding ability of these four fluoroquinolones in the quinolone binding pocket of wild type as well as mutant DNA Gyrase was computationally analyzed by molecular docking to assess their differential binding behaviour. This study has revealed that mutations in DNA Gyrase alter the characteristics of the binding pocket resulting in the loss of crucial molecular interactions and consequently decrease the binding affinity of fluoroquinolones with the target protein. The present study assists in understanding the underlying molecular and structural mechanism for decreased fluoroquinolone susceptibility in clinical isolates as a consequence of mutations in DNA Gyrase.

## Introduction

Drug resistance towards quinolones still remains a major health problem despite the development of newer generation of drugs [1, 2]. The rampant use of fluoroquinolone antibiotics for the treatment of enteric fever has resulted in decreased drug susceptibility as a consequence of acquired resistance in the causative organism *Salmonella enterica* serotype Typhi (*S*. Typhi) [3–5]. The fluoroquinolones are broad spectrum antibacterial agents which are known to primarily target the DNA Gyrase in Gram negative bacteria like *S*. Typhi and the topoisomerase IV (topo IV) in Gram positive bacteria like *Staphylococcus aureus* and *Streptococcus pneumoniae* [6, 7]. The exact mechanism of resistance is not known but it has been found to be closely associated with substitutions in DNA Gyrase in case of *S*. Typhi [8].

DNA Gyrase is universally present in eubacteria and introduces negative supercoils in DNA to relax the topological entanglements during the cellular processes of replication, recombination and transcription [9]. It is a type IIA topoisomerase which like topo IV utilizes ATP to cleave the phosphodiester bond of both strands of DNA [10, 11]. It comprises of two subunits each of Gyrase A (GyrA) and Gyrase B (GyrB) and exists together as an active heterotetramer (GyrA_2_-GyrB_2_). The core cleavage complex consists of N-terminal breakage-reunion domain from GyrA and C-terminal Toprim domain from GyrB in the same heterotetrameric architecture [12, 13]. The quinolones are known to exert their action by interfering with the activity of DNA Gyrase and topo IV [14, 15]. First generation of quinolone drugs (like nalidixic acid) have a narrow spectrum and do not contain the typical quinoline ring or fluorine atom. Subsequent generations of quinolones have, however, increased their range of spectrum and possess the quinoline ring. Basically, all fluoroquinolones contain the same quinoline ring with substituted fluorine at 6^th^ position and different substitutions at 1^st^, 7^th^ or 8^th^ position on the ring. Three generations of fluoroquinolones, second (ciprofloxacin and ofloxacin), third (levofloxacin) and fourth (moxifloxacin), are at present commonly used for the treatment of Gram negative and Gram positive bacterial infections.

The emergence of fluoroquinolone-resistant *S*. Typhi strains in the sub-continent is a growing concern in the treatment of bacterial infection. A number of mutations leading to acquired drug resistance for fluoroquinolones have been analysed in *S*. Typhi [4, 8, 16, 17], *Escherichia coli* [18, 19], *Mycobacterium tuberculosis* [20], *Sterptococcus pneumoniae* [21], *Staphylococcus aureus* [22], *Acinetobacter baumannii* [23], *Bascillus anthracis* [24] and *Helicobacter pylori* [25]. Most of the mutations conferring resistance have been found to be limited to a region of the target enzyme referred to as "quinolone resistance determining region" (QRDR) (S1 Table). This region is highly conserved amongst both Gram negative and Gram positive bacteria and primarily lies in the breakage-reunion domain of GyrA between residues 67–106 [8]. The QRDR is primarily a characteristic DNA binding helix-turn-helix motif comprising two α-helices, α3 and α4, connected by a loop. This region in *S*. Typhi as well as in other bacteria is vulnerable to mutations resulting in decreased susceptibility to flouroquinolone antibiotics.

An in-depth understanding of the role of mutations in acquired resistance to fluoroquinolones and the mechanism of drug action is thus imperative to recognize the underlying cause of decreased drug susceptibility. This requires a detailed analysis of fluoroquinolone interactions with the core cleavage complex of DNA Gyrase. The three-dimensional (3-D) structure of the cleavage complex of *S*. Typhi is currently not reported. However, the structural information of DNA Gyrase cleavage complex from *Staphylococcus aureus* in tetrameric form complexed with DNA and ciprofloxacin (PDB: 2XCT) [13] is available. This cleavage complex adopts a 2-fold symmetry wherein two heterodimers comprising GyrA and GyrB are complexed with DNA. Each heterodimer encompasses a quinolone binding pocket (QBP) to facilitate the binding of quinolones. In the reported structure, two molecules of ciprofloxacin have been observed in the complex bound in a similar manner, one in each heterodimer QBP near the nick at the two DNA strand. It clearly reveals that the QBP comprises of mainly DNA, QRDR of GyrA and part of Toprim domain of GyrB. The floor of the QBP is formed by the α4 helix (residues 81 to 93) of the helix-turn-helix motif of QRDR while the highly conserved residues Lys-Gly-Lys (447–449) of GyrB constitute the roof. Two nucleotides at either side of the DNA nick comprise the two walls where on one side of the nick is purine and on the other side is a pyrimidine base. Similar architecture and mode of binding was also observed in the known structures of topo IV from Gram positive bacteria *Streptococcus pneumonia* [12, 26] and Gram negative bacteria *Acinetobacter baumannii* [27].

Our studies on clinical isolates indicate that the most commonly occurring mutations in the Indian strains are Ser83→Phe83/Tyr83 and Asp87→Tyr87/Gly87. Furthermore, these associated point mutations result in an increase in the Minimum Inhibitory Concentration (MIC) of ciprofloxacin, ofloxacin, levofloxacin and moxifloxacin by many fold as compared to wild type. Previous reports from clinical isolates of *S*. Typhi have focussed only on the identification of resistance conferring mutations and are limited to the molecular typing of DNA Gyrase gene [17]. Though, literature exists on the flouroquinolone binding to mutants from other species, till date information on the molecular basis of drug interaction with *S*. Typhi mutants is lacking. We have identified the mutations contributing to the increased MIC values in clinical isolates from North India and studied the effect of these resistance-inducing mutations on the binding of fluoroquinolones using a computational strategy involving molecular modeling and docking. The model structure of the core cleavage complex has been generated for both wild type and mutants. Four representative drug molecules of successive three generations of fluoroquinolones namely ciprofloxacin, ofloxacin, levofloxacin and moxifloxacin have been evaluated to understand the protein-drug interactions. It has been observed that the substitutions of residues Ser83 and Asp87 in GyrA alter the properties of the binding pocket and adversely affect the interactions with fluoroquinolones. This study correlates the increase in MICs of fluoroquinolones with local conformational perturbations in regions of substitutions in DNA Gyrase with loss of protein-drug interactions. Our results thus establish the molecular basis of quinolone induced drug resistance to *S*. Typhi mutations as detected from MIC values in Indian patients.

## Methods

### Ethics statement

This study was approved by Institute Ethics Committee of All India Institute of Medical Sciences (AIIMS), New Delhi, India (Ref. No.: A-67/4.12.2006). Bacterial isolates used in this study were taken from stock strains obtained from the blood samples of patients at diagnostic laboratory, Department of Microbiology, AIIMS. Ethical committee approved the conduct of this study without written or verbal patient consent as this is a routine laboratory procedure for diagnosis of enteric fever and drug susceptibility analysis.

### Bacterial Strains/ Microbiological Data

**Fig 1:**
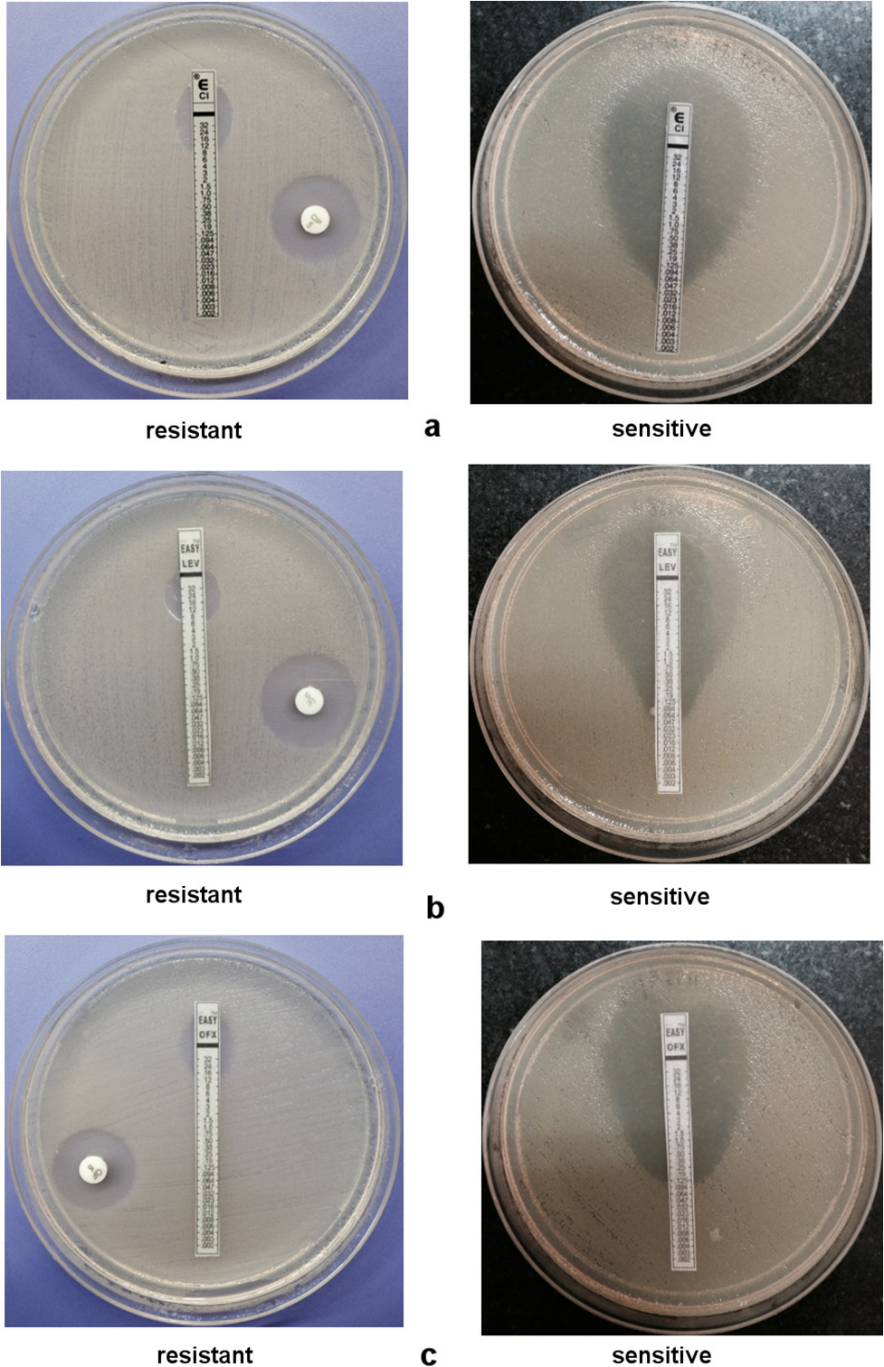
Antimicrobial Susceptibility Test. Susceptibility result of *S*. Typhi by Disc Diffusion method against (a) ciprofloxacin, (b) levofloxacin and (c) ofloxacin.

A total of 100 clinical isolates of *S*. Typhi recovered from blood cultures of patients from the Northern region with enteric fever at All India Institute of Medical Sciences (AIIMS), New Delhi, India were included in this study. These isolates were identified by standard biochemical tests comprising motility, citrate utilization, glucose fermentation, H_2_S production, dulcitol fermentation and decarboxylase reactions. They were serotyped by specific antisera (dH and O9) (Murex Diagnostics Ltd., UK). The antimicrobial susceptibility of the strains was determined by disk diffusion method against ciprofloxacin (5 μg) from HiMedia Laboratories Limited, Mumbai, India according to the Clinical Laboratory Standards Institute (CLSI) guidelines, 2013 [28]. *Escherichia coli* ATCC 25922 and *Pseudomonas aeruginosa* ATCC 27853 were used as reference strains for quality control. The MIC for ciprofloxacin, levofloxacin and ofloxacin was determined using the E-test (AB Bio disk, Solna, Sweden) following the manufacturer’s instructions. The E-test strip of ciprofloxacin contains a predefined gradient of antibiotic concentrations ranging from 0.002 to 32 μg/ml. The dried surface of cation-adjusted Muller Hinton Agar (HiMedia Laboratories Limited, Mumbai, India) plate was streaked uniformly with the test strain using a sterile loop. The E-test strip was placed under sterile conditions and the plate was incubated at 37°C for 16 to 18 h in an inverted position in an incubator under aerobic condition. On incubation an elliptical zone of inhibition was produced and the MIC was read directly from the graduated E test strip at the point of intersection of the zone of inhibition of growth (Fig 1).

MIC for moxifloxacin was determined by micro-broth dilution method [29]. Briefly, micro-broth dilution was carried out in 96-well sterile microtiter plate. Antibiotic solution was prepared in sterile cation-adjusted MHB (Muller Hinton Broth) (Difco Laboratories, Detroit, MI, USA) in sterile test tubes according to the required concentration. As the antibiotic solution is later inoculated with an equal amount of bacteria in broth, the dilutions were prepared at a concentration twice the desired final concentration. To each bacterial isolate, 50 μl of each antibiotic dilution was added into the respective well. Bacterial suspension was adjusted to 1 x 10^8^ cfu ml^-1^ by vortexing and 1:100 dilution was prepared. Then 50 μl of the bacterial suspension was inoculated in each well containing antibiotic dilution resulting in final inoculation concentration of 5 x 10^5^ cfu ml^-1^. Microtiter plate was incubated at 37°C for 16–20 hours. After 16–20 hours, plates were read for MIC. In order for the test to be valid, a definite turbidity or sediment (button size in microtiter plates ≥2 mm), was checked. MIC in a representative number (about 20) of isolates was further confirmed by E-test (AB Bio disk, Solna, Sweden) as described earlier.

### PCR amplification and DNA sequencing of the QRDR

**Fig 2:**
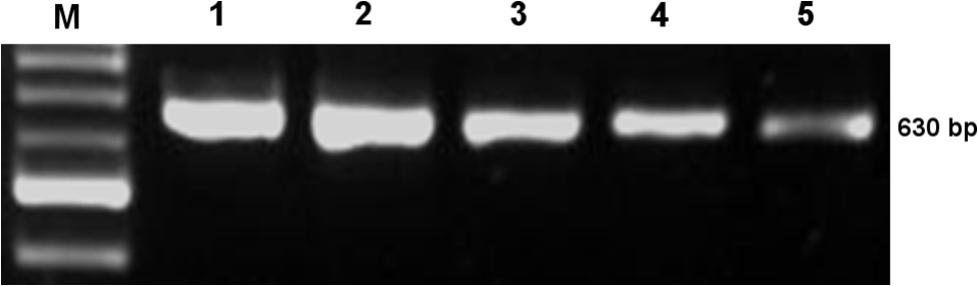
Gel electrogram of PCR product. PCR product of *gyrA* QRDR of *S*. Typhi where Lane M contains the 100 bp ladder whereas Lane 1–5 indicate the product size of 630 bp.

DNA isolation was done by boiling method. Briefly DNA was isolated from overnight bacterial culture equivalent to approximately 10^9^ cells. The culture was centrifuged at 12500 rpm for 5 minutes, pellets were added in 100 μl of sterile redistilled water and vortexed to form a suspension. The suspension was boiled at 100°C for 10 minutes and an equal volume of chloroform: isoamyl alcohol (24:1) solution was added to it. The suspension was then centrifuged at 10000 rpm for 10 minutes. The supernatant containing bacterial DNA was aspirated. Approximately 100 ng of DNA was used in the PCR as template. Amplification of the QRDRs of *gyrA*, *gyrB*, *parC* and *parE* was carried out using published primers earlier reported from our laboratory [4]. The PCR was performed in a final reaction volume of 50 μl containing 5 μl of 10X polymerase buffer and 2.65 U of Taq DNA polymerase. The PCR amplified DNA segment was electrophoresed along with DNA molecular weight marker (100 bp DNA ladder) (Bangalore Genei India Pvt Ltd., India) in 1.5% (w/v) agarose gel (Life Technologies, GibcoBRL, Scotland) prepared in 0.5X Tris-borate ethylenediamine tetra acetic acid buffer (Sigma-Aldrich Pvt Ltd., India). The PCR product was observed after staining agarose gel with ethidium bromide (0.5 μg/ml) by using a ‘ChemiImager Ready’ gel documentation system (Alpha Innotech Corporation, California, USA) and the gel was photographed using Gel Doc (Bio-Rad Laboratories, Hercules, California, USA) (Fig 2).

Sequencing was carried out by the dideoxynucleotide chain termination method using an automated DNA sequencer ABI PRISM 310 Genetic Analyzer (Applied Biosystems, Foster City, California, USA) with AmpliTaq Gold DNA polymerase (Applied Biosystems, Foster city, California, USA) which is a modified form of AmpliTaq DNA Polymerase. Big Dye Terminator v3.1 Cycle Sequencing Kit (Applied Biosystems, Foster City, California, USA) was used for cycle sequencing on ‘Gene Amp PCR system 2400’ thermal cycler (Applied Biosystems, Foster City, California, USA).

### Control DNA sequence

Control DNA, pGEM-3zf and M-13 primer were provided in the ABI PRISM BigDye Terminator Cycle Sequencing Ready Reaction Kit. Cycle sequence reaction and purification procedures for control DNA were similar to that used for sample DNA.

### Sequence Analysis

Reference sequences for all QRDR regions (*gyrA*, *gyrB*, *parC and parE*) were obtained from http://www.ncbi.nlm.nih.gov/gene and compared with the sequence of *S*. Typhi strain CT18 (accession no. AL627274). Sequence chromatograph files were analyzed using BioEdit 5.0.9 [30] to resolve nucleotide ambiguities. Global alignment of the sequences was carried out using the softwares ClustalX 1.8 and GeneDoc 2.1.02 [31].

The QRDR region of target enzymes (*gyrA*, *gyrB*, *parC* and *parE*) was analyzed in one sensitive and all resistant isolates. Mutational alterations were observed in QRDR region of target enzymes in *gyrA* gene only in resistant isolates with MIC ranging from 2 to 8 μg/ml. The mutations observed in these clinical isolates were subjected to a detailed *in silico* study for analysis of the molecular basis of antibiotic resistance.

### Modeling Studies

The 3-D structure of the core complex of wild type as well as mutant *S*. Typhi Gyrase (*st*Gyr) complexed with DNA is necessary to analyse the effect of mutations on the fluoroquinolone binding. In the absence of an experimentally determined structure, a homology model for the complex has been built independently from GyrA, GyrB and DNA fragment. The binding pocket for fluoroquinolones comprises the breakage-reunion domain of GyrA, Toprim domain of GyrB and duplex DNA. The model of breakage-reunion domain of GyrA and Toprim domain of GyrB was generated individually and subsequently the heterotetrameric GyrA_2_-GyrB_2_ complex along with DNA was developed. All the studies have been performed in the modeling environment of Discovery Studio (DS) 2.0 [32].

### Native and mutant homology structures of GyrA and GyrB of *S*. Typhi

The homology model of breakage-reunion domain (residues 30–522) of *st*GyrA was built with the help of MODELLER 8.2 [33, 34]. The homologous structure of *Escherichia coli* GyrA (PDB: 1AB4) [35] with a sequence identity of 93% served as a suitable template for model generation. The examination of the probable differences in side chain conformations in the model is essential as these might vary from the template structure owing to differences in the nature of residues. Therefore, the side chains were refined by the program ChiRotor to obtain the correct conformation [36]. The resulting structure was evaluated for accuracy of stereochemical parameters like conformational angles, bond length, bond angle and G factor utilizing PROCHECK [37] and for structure-sequence relationship using Verify 3D [38]. The model structure was optimized by energy minimization to remove any steric clash with the help of CHARMm force field (version c33b1) [39, 40] and conjugate gradient minimization algorithm [41] with the convergent criteria of root mean square (r.m.s) gradient less than 0.05 kcal/mol/Å.

The Toprim domain model structure (residues 408–628) of *st*GyrB was built, evaluated and optimized in a manner similar to *st*GyrA. The crystal structure of the Toprim domain of *Staphylococcus aureus* GyrB (PDB: 2XCT) [13] with a sequence identity of 64.8% served as the template.

The model structures of the four *st*GyrA mutants (Ser83→Phe83/Tyr83 and Asp87→Tyr87/Gly87) were generated using ‘Build Mutants’ program. This program mutates the selected residue into the specified residue and optimizes its conformation along with the neighbouring residues. The side chain conformations of the substituted residues in the mutant models were also refined with ChiRotor. The stereochemical quality of the models was evaluated by PROCHECK and their structure-sequence relationship assessed by Verify 3D. The model structures of mutant proteins were optimized by energy minimization similar to wild type GyrA. Since no mutations were observed in *st*GyrB in our study, we did not build any mutant. The optimized model structures of GyrA and GyrB were used to develop the core cleavage (GyrA_2_-GyrB_2_)-DNA complex.

### Modeling of GyrA_2_-GyrB_2_-DNA complex

Fluoroquinolones are known to bind in the heterotetrameric assembly state in the presence of DNA. Hence, it was imperative to generate the core complex to carry out the present study. The available crystal structure of core complex of *Staphylococcus aureus* (PDB: 2XCT) served as the framework for the construction of the (GyrA_2_-GyrB_2_-DNA) core complex of *S*. Typhi. The homology models developed for *st*GyrA and *st*GyrB were superimposed onto respective GyrA and GyrB subunits of *Staphylococcus aureus* and its 20-base pair DNA fragment was retained. The constructed structure (henceforth referred as *st*DNA-Gyrase) was energy minimized to remove steric clashes and relax the geometrical constraints produced due to superimposition. The identical protocol was followed to generate the core complex of the four mutants.

### Molecular dynamics simulation of *st*DNA-Gyrase

Molecular dynamics (MD) simulation of the energy minimized *st*DNA-Gyrase was performed with CHARMm (version c33b1) to refine and relax the structure in the presence of TIP3 water model as solvent molecules. Simulations was performed in three stages comprising heating, equilibration and production with time step of 2 fs each and ‘SHAKE' constraints on hydrogen atoms. System was heated to 300K followed by equilibration for 1000 ps and production run for 2000 ps with the help of Leapfrog Verlet dynamics integrator and ‘Berendsen coupling bath’ [42]. The DNA and the protein backbone were kept restrained during the heating and equilibration stages while only DNA was kept restrained with harmonic restraints during the production run.

MD simulation of each mutant complex was performed likewise. These refined complexes were taken as the receptor for docking fluoroquinolones for assessing the consequence of mutations on protein-drug interactions.

### Interactions of fluoroquinolone with *st*DNA-Gyrase

**Fig 3:**
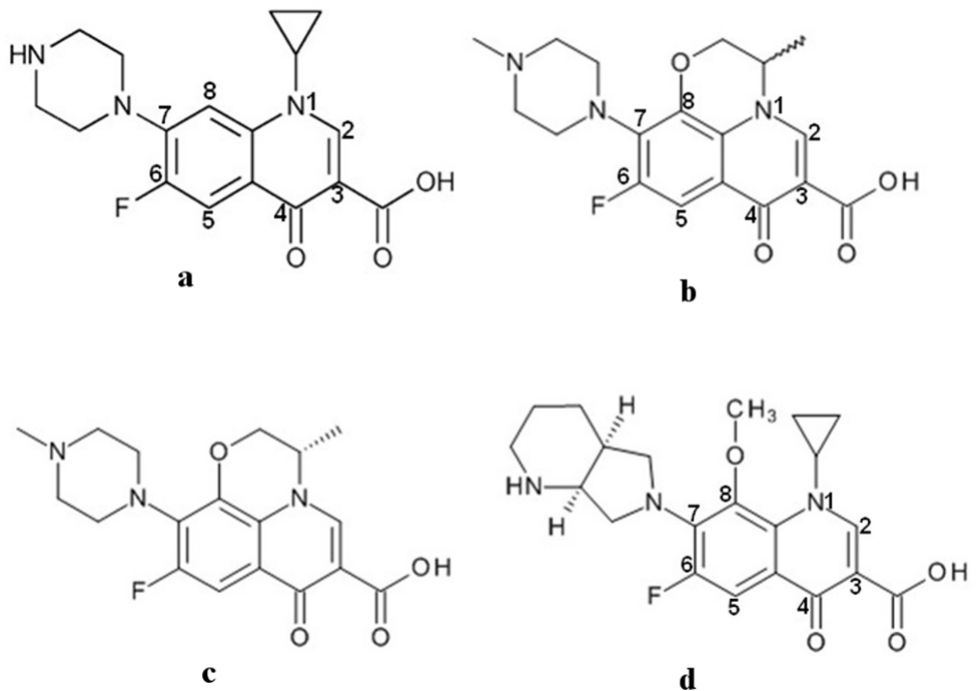
Chemical configuration of quinolones. 2-D chemical structure of second (a) ciprofloxacin and (b) ofloxacin; third (c) levofloxacin and fourth (d) moxifloxacin generation quinolones.

Four commonly used representative fluoroquinolones, two from second generation and one each from third and fourth generation were taken up to determine the molecular mechanism for reduced drug susceptibility. The fluoroquinolones used in this study include ciprofloxacin, ofloxacin, levofloxacin and moxifloxacin (Fig 3). The MIC values were found to be increased in mutants as compared to wild type isolates for all these fluoroquinolones (Table 1). The analysis of the mode of binding and protein-drug interactions are crucial for elucidation of the differential binding behaviour of these fluoroquinolones in wild type and four mutants (Ser83Phe, Ser83Tyr, Asp87Tyr and Asp87Gly) of *st*DNA-Gyrase. This was achieved by individually docking the drugs in their QBP and determining the relative binding strength and nature of interactions to gain insight into the altered binding pattern of fluoroquinolones.

**Table 1:** Minimum Inhibition Concentration (MIC) of different fluoroquinolones and observed mutations in 5 representative strains.

Strain ID	GyrA mutation	Ciprofloxacin(μg/ml)	Levofloxacin(μg/ml)	Ofloxacin(μg/ml)	Moxifloxacin(μg/ml)
1/93	Wild Type	0.032	0.047	0.094	0.032
5432	S83F	3	3	4	3
7999	S83Y	5	5	6	6
15674	D87Y	2	3	4	2
6769	D87G	8	8	8	8

Two molecules of quinolone are known to bind in an almost identical fashion in essentially similar quinolone binding sites. The arrangement and association of the two heterodimers of GyrA and GyrB are due to the presence of a two-fold symmetry in the core complex. Thus, it can safely be assumed that the enzyme-drug interaction of a drug molecule in one QBP can be extrapolated to that for the second molecule. The docking was performed at one binding site only, as the drug-protein interaction observed at one site would hold true for the second site. Hence, the region in the model core complex corresponding to one QBP in one molecule of the dimer was defined as binding site which served as the template for docking of fluoroquinolones.

The co-ordinates of ciprofloxacin (PDB: 2XCT), levofloxacin (PDB: 3K9F), dextrofloxacin (PDB: 2BML) and moxifloxacin (PDB: 3FOF) were obtained from the crystal structures of their respective complexes. The force field parameters of CHARMm were assigned to each structure of the drug molecule, partial charges added based on MMFF94 [43] and subjected to a short run of energy minimization to obtain their low energy conformations. The optimized conformation of each of them was then docked in the defined binding site of *st*DNA-Gyrase using LigandFit [44] docking protocol. LigandFit is a shape based docking program that generates docked poses of ligand with the help of Monte Carlo conformational search method based on the shape of the binding site. The generated docked poses were energy minimized at the binding site and interaction energy between protein-ligands calculated using grid based method. 10000 steps of Monte Carlo conformational search were run to generate the ligand poses. The best 10 docked poses were saved and refined by Dreiding force field followed by scoring with Dock score to rank them. Dock score is a force field based scoring function which is summation of interaction energy and internal energy of the ligand. Docked pose with the highest Dock score (Table 2) were considered as the most probable conformation of the ligand and selected for further analysis.

**Table 2:** Dock Score of four fluoroquinolones for wild type and mutant DNA Gyrase complex of *S*. Typhi.

	**CFX**	**LFX**	**DFX**	**MFX**
**Wild Type**	76.24	78.16	71.43	80.59
**Ser83Phe**	63.14	74.57	64.48	74.40
**Ser83Tyr**	65.83	72.35	64.04	72.62
**Asp87Tyr**	62.35	76.08	68.99	75.28
**Asp87Gly**	59.10	73.95	66.59	71.79

### MD Simulation of *st*DNA Gyrase–fluroquinolone co-complex

In order to assess the stability and probe the pivotal role of the observed hydrogen bonded pattern between Ser83 in wild type *st* DNA Gyrase and quinolone in the stabilization of quinolone ligand, a 100 ns MD simulation of docked complex with a representative quinolone, ciprofloxacin, was performed. The MD simulation for *st*DNA-Gyrase-ciprofloxacin docked complex was carried out by Desmond (v-4.0) simulation package [45] keeping DNA restrained in the presence of SPC solvent model [46] using the OPLS 2005 force field parameters [47]. The dimensions of the cubic simulation box was chosen so that after the protein was placed at the centre of the simulation box, the minimum distance of any protein atom from the edge was 10 Å. The system was neutralized by adding Na^+^ ions to balance the total charge for the system to bring it closer to the physiological environment and ensure a neutral milieu for the system during simulation. Simulations were performed to generate isothermal-isobaric (NPT) ensembles at 300K with Nose-Hoover chain the thermostat method and at 1.01325 bar pressure with Martyna-Tobias-Klein barostat method [48]. Simulations were executed employing the RESPA integrator [49] with a time step of 2 fs and SHAKE constraint [50]. Short range electrostatic interactions and van der Waals interactions were cutoff at a radius of 9.0 Å. The long range electrostatic interactions were calculated by the ‘smooth particle mesh Ewald’ method [51].

## Results

### Determination of MIC and sequence analysis of Gyrase

Overall 100 clinical isolates of *S*. Typhi were taken for this study representing different MIC levels to the four flouroquinolones. All the 100 isolates demonstrated an increase in the MIC values. The antimicrobial susceptibility analysis indicates that 30 clinical isolates were ciprofloxacin resistant, 17 were ciprofloxacin sensitive and 53 were intermediate. All 30 strains resistant to ciprofloxacin also showed resistance to ofloxacin, levofloxacin and moxifloxacin. Hence, the sequence of QRDR of target enzymes (*gyrA*, *gyrB*, *parC* and *parE*) of these 30 resistant and one sensitive isolate (negative control) were analyzed.

The sequence analysis of these isolates revealed point mutations only in *gyrA* of the QRDR region (Table 1). No mutations were detected in the QRDRs of *gyrB*, *parC* and *parE*. This conforms to earlier reports that the mutations conferring 'acquired resistance' are concentrated in the QRDR region of *gyrA* [8, 16]. In mutant GyrA, the amino acid substitution was at (i) residue 83 which was changed from serine to phenylalanine in 14 isolates or tyrosine in 6 isolates and (ii) residue 87 which was mutated from aspartic acid to tyrosine in 7 isolates or glycine in 3 isolates. We found that the single point mutations in *gyrA* are associated with decreased susceptibility to the fluoroquinolones and resistance with an associated increase in MIC of chosen antibiotics. These drug-resistant associated mutations observed in *gyrA* were explored further to investigate the conformational changes induced in the protein as a consequence of mutation and the resultant modifications in the protein-drug interactions which contribute to the alteration in drug susceptibility.

### *S*. Typhi GyrA and GyrB structural models

Homology modeled structure of *st*GyrA reflects a comparatively low r.m.s deviation of 0.38 Å with template structure (S1A Fig) due to the high (>90%) sequence identity between them (S2 Fig). Ramachandran Plot (PROCHECK) obtained for the model with 91% residues in the most favoured region indicates good stereochemical parameters (S2 Table). Verify 3D score (0.07 to 0.67) further validates the sequence-structure compatibility i.e. location of residues (surface exposed or buried, α-helix, β-sheet or β-turn) with their propensity. All these parameters indicate the good quality of the model structure.

Similarly, the model structure of *st*GyrB was comparable to the template with a r.m.s deviation of 0.62 Å (S1B Fig). The Ramachandran plot analysis indicated a good stereochemical quality with 93% of the residues in the most favoured region. Verify 3D score ranging from 0.10 to 0.72 further justifies the compatibility of location and local environment of atomic co-ordinates for each residue with their sequence propensity.

**Fig 4:**
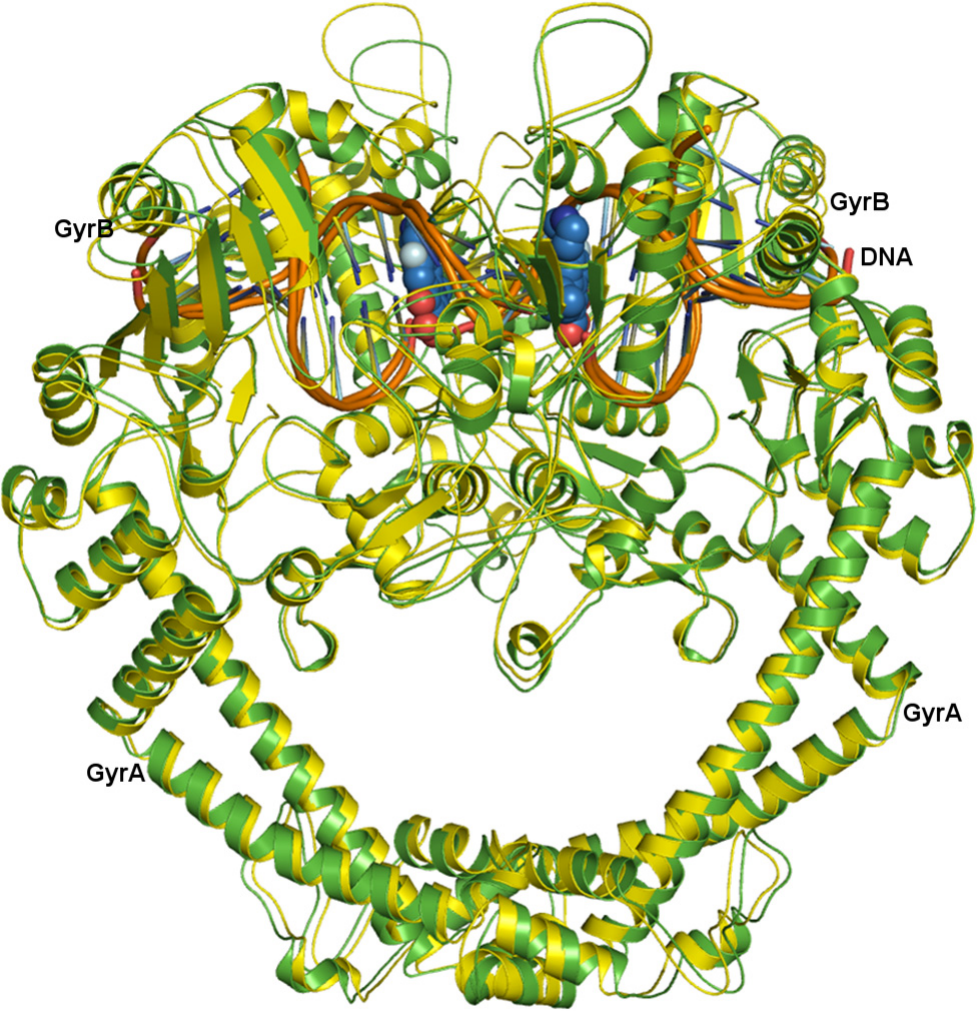
*st*DNA-Gyrase Structure. The homology modeled core complex of *st*DNA-Gyrase (cartoon, green) superimposed on crystal structure of *Staphylococcus aureus* (carton, yellow) with the DNA fragment (orange) and fluoroquinolones (sphere, blue). The structure clearly reveals the two-fold symmetry present in the heterodimer and shows the identical binding of the drug molecule in their respective quinolone binding pocket located in each dimer.

The generated model core complex (GyrA_2_-GyrB_2_-DNA) from individual subunits had a r.m.s deviation of 1.66 Å with the backbone C^α^ atoms of the crystal complex of *Staphylococcus aureus* DNA Gyrase (PDB: 2XCT) with minor variations in the loop region located away from the QBP (Fig 4). The overall architecture of the *st*DNA-Gyrase adopts a structural organization similar to that reported for other bacterial type IIA topoisomerases [12, 13, 26, 27].

**Fig 5:**
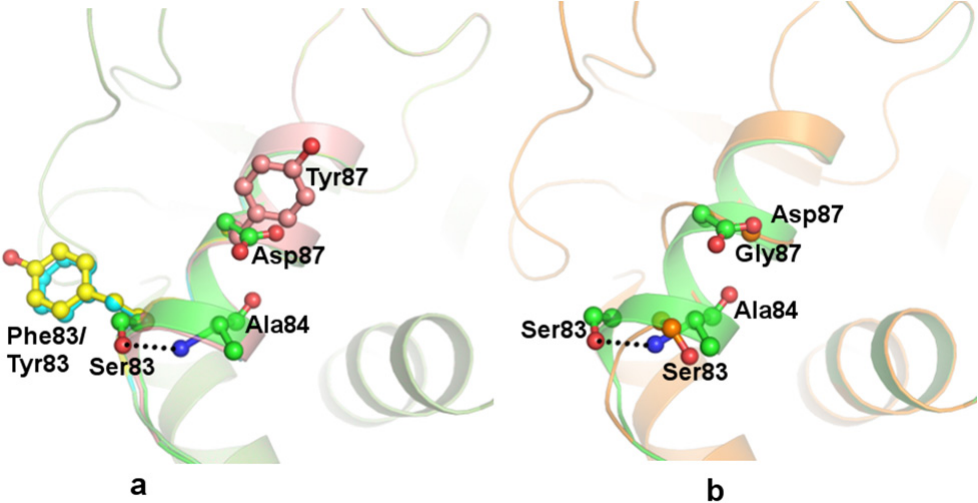
Wild type and mutant *st*GyrA. Model structure of wild type *st*GyrA (cartoon, green) superimposed on mutants showing side chains in ball and stick to indicate the differences in the wild type and mutant structures at the site of substitution (a) Ser83Phe (cyan), Ser83Tyr (yellow), Asp87Tyr (brick) and (b) Asp87Gly (orange).

The QBP is located at the nick in DNA and the aromatic quinoline scaffold intercalates between two immediate bases at either side of the nick. The carboxylate moiety of quinolone occupies the part of the QBP formed by GyrA. The most widely observed mutations in quinolone resistant strains are harboured in the helix α4 of the helix-turn-helix DNA-binding motif in the QRDR of GyrA adjacent to the nick. Likewise, all the mutations observed in this study from the clinical isolates are housed on helix α4 of this helix-turn-helix motif comprising the α3 and α4 helices. The model structures of wild type and mutant proteins (Ser83Phe and Ser83Tyr) are similar except for minor variations in the loop regions near the site of mutations. Ser83 is a small and polar residue and forms the floor of QBP. It is the most commonly observed site of mutation indicating its prime importance for quinolone activity. The position of hydroxyl group of Ser83 side chain is stabilised by hydrogen bonded interaction with amide group of Ala84 in wild type protein (Fig 5). The substitution of Ser83 by aromatic residues, Phe or Tyr, leads to the introduction of residues with a longer and bulkier side chain. These variations in the mutants induce local perturbations in the protein backbone. The bulkier aromatic side chains of Phe/Tyr introduce steric hindrance and cannot be accommodated in the same direction as the much smaller Ser. This results in the reorientation of the Phe/Tyr side chains (Fig 5A) and subsequently alters the geometry and binding characteristics of the QBP. Similarly, the bulkier Tyr87 in mutant Asp87Tyr projects in a different orientation compared to Asp87 and consequently affect the shape and size of the QBP. The maximal change in the protein structure is induced by the replacement of Asp87 with Gly. In the mutant Asp87Gly, the smaller Gly residue comprises a hydrogen atom as the side chain. This substitution modifies the attributes of the binding pocket by enhancing the flexibility of the protein backbone locally. This leads to the disruption of the helical conformation in the protein from residues Ser83 to Thr88 (Fig 5B). As a consequence, the change induced in the size and shape of the binding pocket is more pronounced resulting in decreased binding efficacy which is reflected in the higher MIC values obtained for the clinical isolates.

### MD simulations of *st*DNA Gyrase model complex

MD simulation was performed in the presence of solvent molecules for the final refinement of the complex. Production run of 2 ns after the equilibration phase of 1 ns was performed. The complex converged at 600 ps of production phase of simulations. The potential energy of the resultant complex was stable with insignificant fluctuations. The r.m.s deviation of the model complex with respect to the initial conformation was in the range of 2 to 3 Å (S3 Fig) indicating a stabilized and relaxed structure. This refined model complex was taken up for the docking study of fluoroquinolones.

### Gyrase-fluoroquinolones interactions

The determined MICs in this study clearly reveal that the fluoroquinolones are less effective against the isolates with mutated GyrA. Therefore, to shed light on the molecular mechanism behind the disparity in the drug activity, molecular docking investigation of the fluoroquinolones was performed in the QBP of *st*DNA-Gyrase of wild type and mutants to evaluate the association of quinolone resistance and mutation. The docked conformation of ciprofloxacin in *st*DNA-Gyrase was observed to be similar to its bound conformation in *Staphylococcus aureus* co-crystal complex, thus validating the docking protocol (S4 Fig). The position and orientation of quinoline ring, fluorine atom and the attached moieties (like the cyclopropane) are comparable. The quinoline ring is planar and intercalates into DNA to interact with the nitrogenous bases at the nick through stacking interactions, its carboxylate group interacts with Ser83 of GyrA while aza substituent at C-7 position interacts with Lys447 of GyrB. The docked conformation of ofloxacin, levofloxacin and moxifloxacin in the wild type are analogous to ciprofloxacin. However, docking with the mutant complex (Ser83Tyr/Phe and Asp87Tyr/Gly) revealed differences in their binding with the loss of crucial hydrogen bonded interactions with the fluoroquinolones. The hydrogen bond in the docked complex between Ser83 in the wild type protein and ciprofloxacin was stable despite small fluctuations in the position of ciprofloxacin during simulation.

## Discussion

### MIC of quinolones and association with mutation in clinical isolates of *S*. Typhi

The quantitative determination of the MIC values derived from clinical isolates from Indian patients reveals decreased drug susceptibility towards various fluoroquinolones suggesting the emergence of resistant strains. MIC values varied from 0.032 to 8 μg/ml and there was no significant difference within the three successive higher generations of quinolones. The estimated MIC indicated that 30% of total clinical isolates in this study were resistant to ciprofloxacin, ofloxacin, levofloxacin and moxifloxacin according to latest Clinical and Laboratory Standards Institute (CLSI) guidelines. Mutations were observed only in *gyrA* gene and were not found in *gyrB*, *parC* or *parE*. Sequence analysis of these 30 resistant strains has indicated mutation at residues Ser83 and Asp87 in GyrA. Mutation at Ser83 was more frequent than Asp87 with a 2:1 ratio. Substitution of Ser83 with Phe83 was most common with 70% cases of mutation at this position. The extremely high prevalence of replacement of residue corresponding to Ser83 has also been observed in Gram negative bacteria *Escherichia coli* [52] and Gram positive bacteria *Bacillus anthracis* [53]. Mutational screening in majority of such investigations has reported single point mutations in *gyrA* to be associated with decreased drug susceptibility. Earlier investigations from the subcontinent have reported resistance conferring mutations in *S*. Typhi to be associated with substitutions at positions 83 and 87 [54]. Studies carried out in neighbouring countries have also indicated a similar trend of development of antibiotic resistance. Similar pattern of resistance-mutation association was found in patients from Hong Kong [16], Cambodia [55] and neighbouring Nepal where alterations in GyrA with sporadic incidences of *parC* mutations have been reported [56]. In addition to these amino acid replacements, the drug resistance in *S*. Typhi has also been attributed to an altered drug efflux mechanism [57, 58]. This particularly gains significance where there is clinical failure to respond to treatment by fluoroquinolones in enteric fever. A study conducted previously to evaluate the role of the efflux pump mediated mechanism towards ciprofloxacin resistance revealed that the efflux mechanism does not have a role in these resistant clinical isolates [58]. The mutational screening conducted, however, revealed that the point mutations in the QRDR of *gyrA* probably play an important role in drug resistance.

### Protein-drug interactions in cleavage complex

The fluoroquinolones exert their action through stabilization of the Gyrase—DNA cleavage complex. DNA Gyrase acts by cleaving the phosphodiester bond via a nucleophilic attack and the catalytic Tyr122 residue from GyrA to form a transient covalent bond with 5'-phosphate of DNA after cleavage. Ligation of the cleaved strand is restored after removal of strains due to supercoiling. Binding of quinolone stabilizes this complex in the broken state of DNA and inhibits the resealing of the break. The arrest of resealing of the breaks thereby halts the process of replication and transcription, consequently killing the bacteria. The mode of interaction is revealed from the co-crystal complex of wild type DNA Gyrase with ciprofloxacin [13], topo IV with levofloxacin [26] and moxifloxacin [27] which showcase the important drug-protein interactions. The quinolones are planar molecules and intercalate into the DNA between aromatic nitrogenous bases at the nick. The binding of quinolone is stabilized by stacking interactions of its quinoline ring with aromatic base of DNA from each side of the nick whereas its carboxylate moiety interacts with residues residing on the α4 helix of the helix-turn-helix motif in the QRDR. Mg^2+^ has been observed near C3-C4 keto acid of ciprofloxacin in Gyrase [PDB: 2XCT] and moxifloxacin in topo IV [PDB: 2XKK]. Mg^2+^ in the native protein is believed to be required for quinolone action [59, 60] and it has been shown that this ion is stabilized through water mediated interactions with Ser84 (Ser83 in *S*. Typhi) and Glu88 (Asp87 in *S*. Typhi) in *Acineatobacter baumannii* [27]. However, Mg^2+^ ion was not observed in topo IV of *Streptococcus pneumoniae* [PDB: 3K9F] where the carboxylate group of levofloxacin was within hydrogen bonded distance with Ser residue. Furthermore, the available crystal structures of quinolones complexed with DNA Gyrase from S. *aureus* (PDB: 2XCT) and topo IV (PDB: 3K9F, 3RAD, 3RAE & 2XKK) were also examined for the hydrogen bonded interaction pattern between Ser and quinolones. Overall, these co-crystal structures exhibit the presence of hydrogen bonded interactions between quinolones and the Ser residue of wild type DNA-Gyrase (corresponding to Ser83 in *st*DNA-Gyrase). Moreover, this hydrogen bond is observed irrespective of the presence or absence of Mg^2+^. The consistent occurrence of this intermolecular hydrogen bond in all these structures clearly signifies that it plays a key role in quinolone binding. Therefore, it might be possible that the direct interaction between Gyrase and quinolone could be playing a significant role along with water mediated stabilization of Mg^2+^. This direct role of drug-protein interactions have been investigated in this study.

### Binding of drug with wild type *st*DNA-Gyrase

The co-crystal complexes of type IIA topoisomerases with fluoroquinolones reveal the mode of their recognition to DNA Gyrase [13, 26, 27]. The antibacterial agent is known to strongly bind to the Gyrase-DNA complex whereas it can bind only weakly to the individual protein or DNA. The fluoroquinolone intercalated between nucleotides also interacts with residues on the helical region of QRDR of GyrA in addition to DNA and GyrB. The binding of quinolone is stabilized by stacking interactions between aromatic nitrogenous base of DNA and quinoline ring. Furthermore, hydrogen bonded interactions are present between carboxylate group of quinolones and surrounding residue Ser83 in QRDR of GyrA. The Ser83 resides on the helix-turn-helix motif in GyrA and is hydrogen bonded with carboxylate moiety of quinolone whereas the highly conserved residues Lys-Gly-Lys (447–449) of GyrB interacts with substituent at the C-7 position of the drug through van der Waals interactions. The direct role and stability of drug-protein interactions mediated via the hydrogen bonded pattern is evident in this study by the 100 ns MD simulation performed for the *st*DNA Gyrase—ciprofloxacin docked complex. The existence of the hydrogen bond during simulation suggests that the hydrogen bonded interaction mediated through Ser83 in protein-drug complex (or the corresponding Ser residue) contributes majorly to the stabilization of the binding of quinolone to wild type DNA Gyrase. Hence, the disruption of this hydrogen bond dependent drug interaction will lead to fluoroquinolone resistance.

### Mutation induced conformational alteration in *st*DNA-Gyrase

The most commonly observed mutation in resistant *S*. Typhi concerns the residue Ser83. Ser83 in *st*GyrA and its corresponding residue in Gyrase or topo IV in other bacteria have been found to be crucial for fluoroquinolone action. This residue is indicated to confer antibacterial activity towards these drugs. Its replacement in the mutants, leads to increase in the MIC value for quinolones. The Ser residue (Ser84 of ParC in *Acinetobacter baumannii*) reportedly stabilizes the proper positioning of Mg^2+^ through water mediated interactions and has been indicated to be essential for quinolone activities [27]. The substitution of Ser83 in the two mutants in DNA Gyrase by Phe/Tyr introduces residues with bulkier and relatively hydrophobic side chains which alter the shape, size and electrostatic environment of the quinolone binding pocket. This modification in the mutants leads to the elimination of crucial conserved water molecules stabilized by Ser83 hydroxyl side chain and Asp87 carboxylate in the wild type resulting in the loss of the Mg^2+^ ion stabilized by these water mediated interactions. Another mutation observed in this study associated with increased MIC of fluoroquinolones concerns the replacement of Asp87 by Tyr/Gly. This is the second most frequently associated mutation in resistant isolates of *S*. Typhi. Asp87 projects in the QBP and like Ser83 might be playing a key role in retaining the crucial water molecules involved in Mg^2+^ ion preservation [27]. The bulky aromatic residue Tyr in the Asp87Tyr mutant perturbs the volume whereas its phenolic hydroxyl group alters the electrostatic nature of the binding pocket. The mutant Asp87Tyr due to its longer size displaces the conserved water molecules and decreases the accessible area within the pocket. In the second mutant Asp87Gly, the residue Gly lacks a side chain and imparts flexibility to the protein backbone resulting in the disruption of the typical α-helical conformation. The introduction of Gly also affects the conformation of the critical residue Ser83. Consequently, the substitutions in addition to modifying the geometric properties of the binding pocket also disturb the water molecules essential for quinolone activity.

### Effects of mutation on drug binding

The mutations in *gyrA* of *st*DNA-Gyrase alter the local environment of the protein, decrease drug-protein interaction and prevent the appropriate binding of the drug. This reduces the binding affinity and increases resistance to the drug. Structure-based *in silico* analysis of the drug-protein interaction of wild type and four mutants has been performed employing a computational strategy of homology modeling followed by molecular dynamic simulation and docking to evaluate the relationship between the differential nature of amino acid and drug resistance as indicated by determined MIC of four fluoroquinolones. Hence, the various fluoroquinolones undertaken in this study were individually docked in the MD simulated homology models of the native and four mutant *st*DNA-Gyrase complexes and their mode of binding analyzed in an effort to comprehend the drug acquired resistance in the mutants isolated from the resistant bacterial strains of North Indian patients.

### Ciprofloxacin-*st*DNA Gyrase interaction

**Fig 6:**
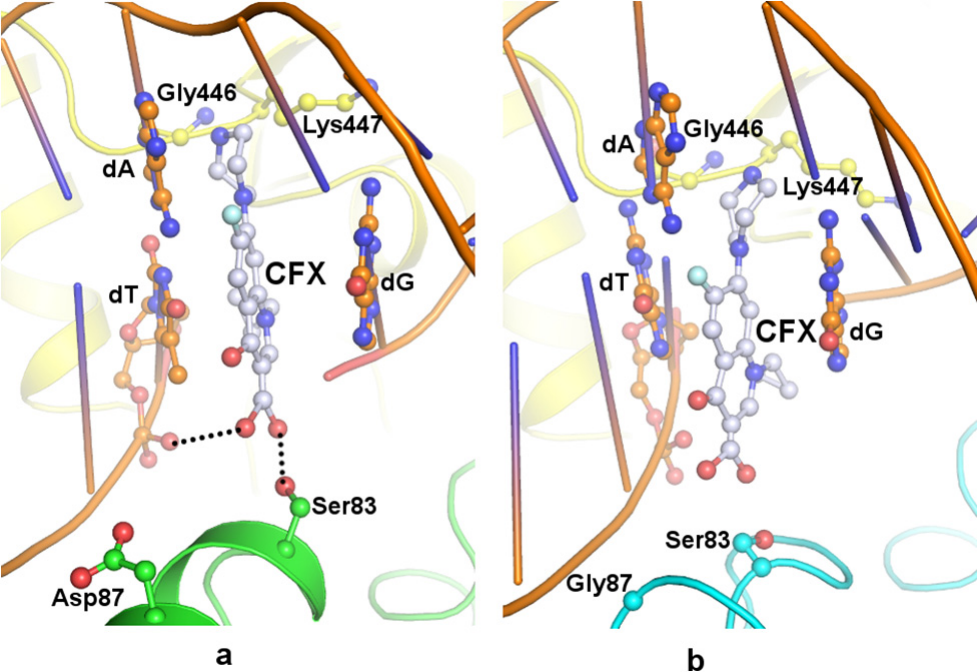
*st*DNA-Gyrase—ciprofloxacin. Docked position of ciprofloxacin (ball and stick, grey) in the QBP of *st*DNA-Gyrase (cartoon) where DNA is drawn in orange, GyrB in yellow (a) wild type GyrA in green and (b) Asp87Gly GyrA mutant in cyan. Ser83 and Asp87/Gly87 are represented in ball and stick in respective colour. Hydrogen bonds are indicated as black dotted lines.

Ciprofloxacin though a second generation quinolone is still a regularly used antibiotic. MIC of ciprofloxacin determined in this study indicated the increased range for mutants in comparison to wild type (Table 1). This fluoroquinolone is smaller in size compared to the other three (Fig 3) owing to a smaller substituent at N-1 or C-7 position. The docked conformation of ciprofloxacin in *st*DNA-Gyrase is similar to its crystal conformation in *Staphylococcus aureus* (PDB: 2XCT). The orientation and position of fluorine atom and cyclopropane moiety of ciprofloxacin are akin to the respective moieties in crystal conformation. Ciprofloxacin was seen to occupy the quinolone binding site and sit at the four base pair staggered nick in the DNA binding groove (S5A Fig). Its aromatic ring intercalates between the bases of two successive nucleotides of the same polynucleotide strand where the drug is sandwiched between the guanine and thymine ring stabilized by π – π stacking interactions. The carboxylate group of ciprofloxacin forms a hydrogen bond with hydroxyl group of Ser83 from GyrA and the phosphate group of nucleotide backbone (Fig 6A). Retention of the hydrogen bond during the simulation manifests its significant role in protein-drug interaction.

The docked conformation of ciprofloxacin in Phe83/Tyr83 mutant is overall similar to wild type. The orientation of substituents like cyclopropane and fluorine is also comparable. The replacement of Ser83 by bulkier residue Phe83/Tyr83 in mutant forces away the carboxylate group of the drug by rotation due to steric hindrance (S5BC Fig). As a result the hydrogen bonded interactions observed in the wild type can no longer be formed. An overall loss of interaction energy occurs between ciprofloxacin and mutant gyrase as compared to wild type. In addition, these mutations also lead to loss of crucial water molecules required to retain the magnesium ion and thus may contribute to the decrease in susceptibility to the drug.

In mutant Asp87Tyr, the bulky side chain of Tyr87 induces modification in the conformation of Ser83 side chain consequently taking it farther from the carboxylate group of ciprofloxacin (S5D Fig) and as a result disturbs the hydrogen bonded interaction between them. The change in orientation of Ser83 combined with the bulky size and aromatic nature of Tyr side chain do not permit the retention of crucial water molecules required for proper positioning and retention of Mg^2+^. This cumulative effect decreases the binding affinity of ciprofloxacin for this mutant. Disruption of helical structure in mutant Asp87Gly alters the position and orientation of Ser83 so that it is no longer within hydrogen bonding distance with the drug carboxylate group (Fig 6B). Additionally, this causes the loss of van der Waals interactions between ciprofloxacin and enzyme as indicated by lower steric energy compared to other three quinolones. Change of side chain conformation of Ser83 might also affect position of water molecules required for stabilization of Mg^2+^.

The orientation of ciprofloxacin in the binding pocket of native and mutant enzyme is similar except for minor variations in the position of the ligands. The bulkier side chains in the mutants force the ciprofloxacin away from GyrA. The mutants lack the capacity to retain the critical hydrogen bond between ciprofloxacin and enzyme in addition to loss of water mediated interactions with Mg^2+^ required for quinolone action. Overall, this weakens the enzyme-drug interactions. The decrease in the calculated interaction energy for mutants is comparable to the higher IC_50_ values of ciprofloxacin present in the closely related DNA Gyrase from *Escherichia coli* (wild type = 4.6 ± 0.21 mg/L, Ser83Leu = 8.62 ± 0.68 mg/L & Asp87Gly = 13.1 ± 2.78 mg/L) [61].

Therefore, this modeling-based molecular recognition of the drug suggests that the variation in the drug-protein interactions owing to mutations at Ser83 and Asp87 might be responsible for the resistance against ciprofloxacin. The calculated interaction energies also indicate that Ser83Phe and Ser83Tyr mutations have a more pronounced effect on ciprofloxacin resistance than Asp87Tyr. This is due to the proximity of residue 83 to quinolone in comparison to residue 87 in the QBP and the presence of direct hydrogen bonded interaction between residue 83 and ciprofloxacin in addition to destabilisation of water mediated interactions with Mg^2+^. However, the most deleterious effect has been observed due to the Asp87Gly mutation wherein it induces a conformational modification in the region housing the Gly residue leading to loss of direct hydrogen bond between drug and Ser83 along with van der Waals interactions with surrounding residues.

### Levofloxacin-*st*DNA Gyrase interaction

Levofloxacin, a third generation fluoroquinolone, is one of the most effective antibacterial drugs to be used in the treatment of uncomplicated enteric fever. Docking of levofloxacin revealed its location in the quinolone binding pocket to be comparable to ciprofloxacin (S6 Fig). The two compounds differ in their substituent at N-1 and C-8 positions wherein the region of QBP occupied by cyclopropyl moieties of ciprofloxacin is engaged by the methylated cyclic ring of levofloxacin. The intercalated quinoline moiety of levofloxacin is sandwiched between the guanine and thymine ring of the nucleotide at the nick like ciprofloxacin. The fluorine atoms in both these drugs are situated at similar sites.

The carboxylate group in both drugs forms hydrogen bonded interactions with the phosphate group of nucleotide and hydroxyl group of Ser83 in wild type (S6A Fig). The substitution of Ser83 by bulky residues Phe83/Tyr83 in mutants forces the carboxylate moiety of levofloxacin away from GyrA and causes the loss of the hydrogen bond with enzyme and nucleotide phosphate in a manner analogous to ciprofloxacin (S6BC Fig). This is supported by the decline in interaction energy that eventually leads to reduction in binding strength of levofloxacin for mutants in comparison to native gyrase.

Binding of levofloxacin to the mutant Asp87Tyr was also found to be affected. The steric constraints introduced due to the bulkier Tyr87 induce the reorientation of the Ser83 side chain and disrupt the hydrogen bond with levofloxacin (S6D Fig). This hydrogen bond is also lost in the Asp87Gly mutant due to conformational modification in the QBP (S6E Fig). This decreased calculated interaction energy indicates a diminished binding strength and corroborates the experimental MIC value. Hence, the hydrogen bond existing between the protein and drug molecule might be crucial for the pharmacological activity of drug and its absence leads to a reduction in susceptibility of pathogens for levofloxacin.

### Ofloxacin-*st*DNA Gyrase interaction

Ofloxacin, another commonly employed antibacterial drug is a racemic mixture comprising equal proportions of levofloxacin and dextrofloxacin. Dextrofloxacin differs from levofloxacin at the configuration of methyl substituent on cyclopentyl ring (Fig 3). Since there is no fixed configuration of ofloxacin, the enantiomeric form of dextrofloxacin has been used for docking study with *st*DNA-Gyrase and its calculated activity has been averaged with that of levofloxacin. In the wild type, like levofloxacin, the carboxylate group of dextrofloxacin forms hydrogen bonded interactions with hydroxyl group of Ser83 and phosphate backbone of DNA (S7A Fig). The orientation of methyl substituent in this enantiomeric form results in steric hindrance owing to van der Waals clashes with nucleotide and decreases the interaction energy in contrast to levofloxacin. The substituted residues Phe/Tyr83 in mutant forces away the carboxylate moiety of dextrofloxacin in a manner identical to the levo form and disturbs the hydrogen bond formed with Gyrase and nucleotide (S7BC Fig). The binding conformation and affinity of dextrofloxacin with Asp87Tyr (S7D Fig) and Asp87Gly (S7E Fig) mutants resembles that of levofloxacin.

### Moxifloxacin-*st*DNA Gyrase interaction

Moxifloxacin is a current antibiotic of the fourth generation in practice for antibacterial treatment. The docking of moxifloxacin indicated its conformation in wild type to be comparable to that in topo IV of *Acinetobacter baumannii* (PDB: 2XKK) as well as similar to the docked ciprofloxacin. The cyclopropane moiety of the intercalated drug like ciprofloxacin interacts with the first base while C-7 substituent bicyclic diaza moiety forms van der Waals contacts with base of the opposite strand and long alkyl chain of conserved Lys447 of GyrB (S8A Fig). Similarly, the carboxylate group of moxifloxacin interacts with nucleotide and protein through hydrogen bonds.

The replacement of Ser83 by Phe83/Tyr83 in mutants leads to loss of hydrogen bond between moxifloxacin and GyrA as well as with DNA like the other three quinolones studied (S8BC Fig). The larger Tyr87 in Asp87Tyr mutant reduces the size of the QBP causing the readjustment of the Ser83 hydroxyl side chain and does not permit the hydrogen bond formation with moxifloxacin (S8D Fig). This is in agreement to that observed for the other three quinolones under consideration. Likewise, in mutant Asp87Gly, the hydrogen bond between protein and moxifloxacin was absent due to the disrupted helical conformation and reorientation of Ser83 (S8E Fig). The calculated interaction energy of moxifloxacin is equivalent to the IC_50_ values observed for *Escherichia coli* DNA Gyrase (wild type = 2.93 ± 0.38 mg/L, Ser83Leu = 3.35 ± 0.4 mg/L & Asp87Gly = 4.4 ± 0.55 mg/L) [61]. This clearly verifies the mechanism of resistance explained by this modeling study.

### Overall effect of observed mutations on drug susceptibility to fluoroquinolones in *st*DNA-Gyrase

**Fig 7:**
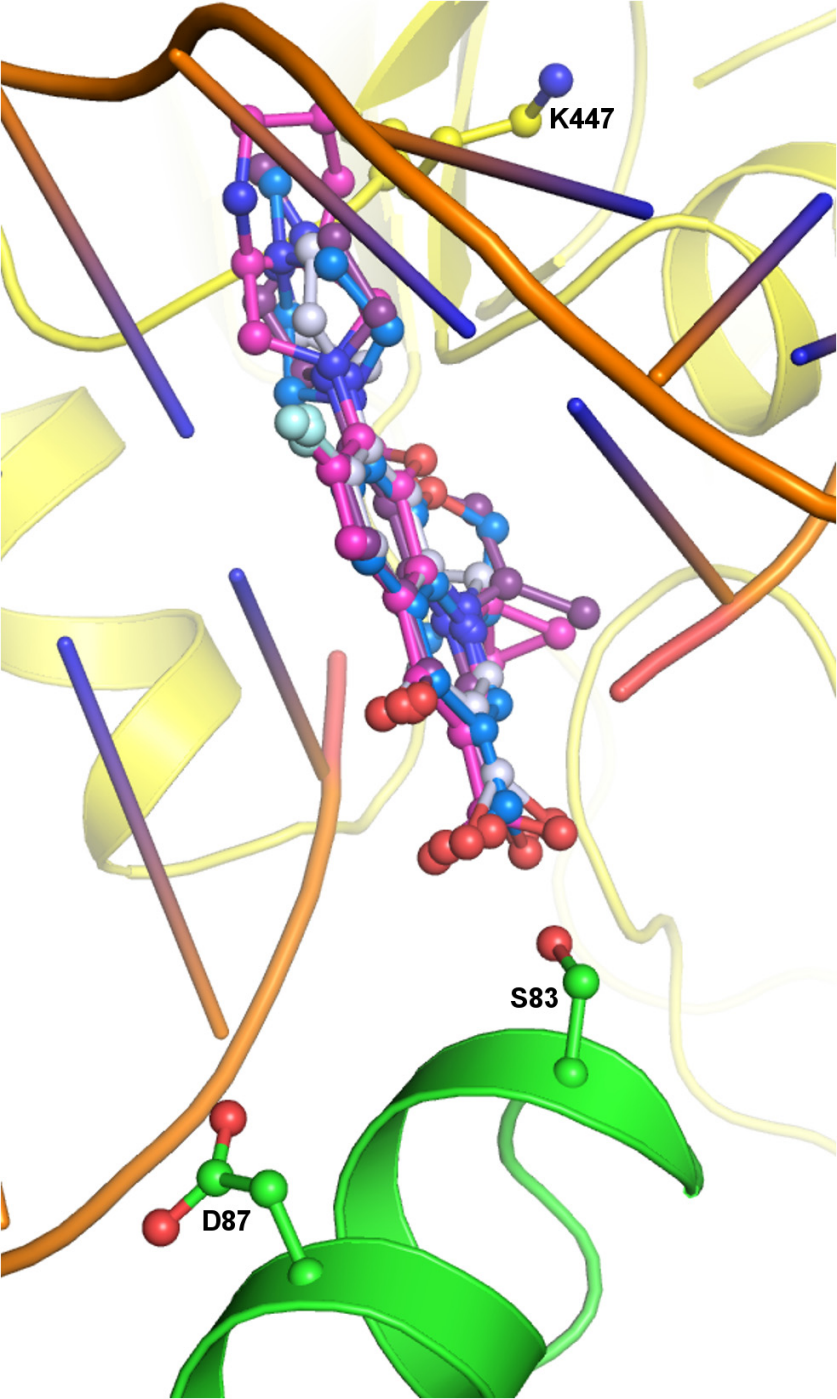
Superimposition of quinolones. Docked conformation of ciprofloxacin (grey), levofloxacin (blue), dextrofloxacin (purple) and moxifloxacin (magenta) indicated in ball and stick in the QBP of *st*DNA-Gyrase. DNA is shown as cartoon (orange) and proteins with side chain of Ser83 and Asp87 (ball and stick) in respective colours.

The diverse induced mutations in *st*DNA-Gyrase occur in the QBP at the α4-helix of the DNA binding motif on which the DNA is stacked. These mutations alter the conformational properties of QBP by changing the shape and size of this pocket and ultimately affect the efficiency of drug binding. The molecular and experimental inhibition analysis in this study clearly reveals that binding mode of the fluoroquinolones in QBP is similar irrespective of different generations of quinolones. The superimposition of docked complexes of ciprofloxacin, levofloxacin, dextrofloxacin and moxifloxacin with native Gyrase indicate comparable positions of quinoline ring and fluorine in the QBP with minor variations owing to different substituents on the quinolone ring (Fig 7) similar to crystal structures [12, 26, 27]. The drugs intercalate into the DNA as a result of a breakage of the DNA strand and interact with the nucleotides. This complex is further stabilized by π-π stacking interactions and van der Waal forces. In all four complexes, quinolones directly interact with Ser83 of *st*GyrA and indirectly interact with Asp87 through water-mediated interactions. Hence, the hydrogen bonded interactions contribute extensively in the drug binding to the native protein. MD simulation of the docked complex also substantiates the importance of hydrogen bond between quinolone and Ser83. This implies that in the wild type, the susceptibility to quinolones in different generations with regard to target interactions is comparable. The point mutations at Ser83 were observed to have a more pronounced effect on drug resistance than those at Asp87. This is a consequence of the direct and indirect role played by Ser83 where it interacts with quinolones through hydrogen bond formation and is required for Mg^2+^ stabilization. In contrast Asp87 is only engaged in the water mediated conservation of Mg^2+^. Our modeling and docking study of each individual mutants (Ser83Phe/Tyr and Asp87Tyr/Gly) with different quinolone derivatives (ciprofloxacin, dextrofloxacin, levofloxacin and moxifloxacin) indicate a shift in the position of quinolones due to loss of this hydrogen bond and a decrease in interaction energy as compared to wild type. The decrease in the interaction energy in the mutants points towards the reduced stability of drug binding. The study demonstrates the vital role of hydrogen bonded interaction between DNA Gyrase and quinolone in the wild type protein. Furthermore, the disruption of this hydrogen bond will result in loss of interaction energy and subsequently decrease in susceptibility to the fluoroquinolones. This explains the associated level of increase in MIC as a consequence of these mutations. This observation validates the role of the loss of crucial hydrogen bond between GyrA and quinolones in the enhancement of their MIC value. Overall, the absence of the hydrogen bond in the mutants coupled with the experimental increase in MIC values points towards the key contribution of hydrogen bonded interaction pattern in quinolone binding.

### Comparison with drug-resistant mutations in other species

The most common site of mutation in either DNA Gyrase or topo IV is the residue corresponding to Ser83 in GyrA of *S*. Typhi followed by acidic residue (Asp or Glu) four residues apart. These residues are highly conserved in DNA Gyrase of both Gram positive and Gram negative bacteria. Approximately 90% of resistant strains from clinical as well as laboratory source have mutation at this position in Gram negative bacteria like *Escherichia coli* and Gram positive bacteria like *Bacillus anthracis* [52, 53]. These two residues lie on the α-helix that partially forms a wall of the quinolone binding pocket. The mutation in GyrA of *Bacillus anthracis* at Ser81 and Glu85 leads to decreased affinity of topo IV for ciprofloxacin [59, 60]. The only available crystal structure of wild type cleavage complex of DNA Gyrase from *Staphylococcus aureus* reveals that Ser84 of GyrA lies in close vicinity (< 4 Å) of non-catalytic Mg^2+^ and carboxylate moiety of ciprofloxacin [PDB: 2XCT]. Similarly corresponding Ser residue of topo IV has been seen to lie in close vicinity to carboxylate moiety of levofloxacin and moxifloxacin as observed in *Streptococcus pneumoniae* and *Acinetobacter baumannii* [PDB: 3K9F & 2XKK]. These structures together with 100ns MD simulation of the *st*DNA Gyrase-ciprofloxacin complex further validate the relevance of hydrogen bonded interaction in protein-drug interaction.

## Conclusion

A number of drug-resistant *S*. Typhi strains have emerged due to the injudicious use of fluoroquinolone antibiotics. These resistant strains from clinical isolates were cultured and the MIC values of four commonly employed quinolones from second (ciprofloxacin and ofloxacin), third (levofloxacin) and fourth generation (moxifloxacin) determined against them. The observed MIC values indicate a reduction in the drug-susceptibility for the mutant bacterial strain which was evaluated with associated mutations in the target DNA Gyrase. Overall the drugs bound at the QBP and adopted similar position and orientation in both wild and mutants. The observed replacement was present only in the QRDR region of the α4-helix of DNA binding motif in GyrA exclusively at Ser83 and Asp87 residues. These two mutations have been observed for DNA Gyrase from different species and regions. Though the binding pocket for quinolones is primarily formed by nucleotides, the residues at positions 83 or/and 87 directly or indirectly interact with the carboxylate moiety of quinolone. The molecular modeling study reveals that the substitutions in the mutant protein lead to local perturbations in the protein structure and alter the chemical environment and geometry of the QBP in terms of both shape and size. The bulkier Phe/Tyr residues in the mutants decrease the overall space within the cavity whereas the supple Gly residue disrupts the helical conformation of α4-helix and alters its geometry. In either case the residue at position 83 is pushed out to accommodate it in the changed environment of the QBP. The docking study of fluoroquinolones with the generated model complex of DNA Gyrase for wild type and mutant *S*. Typhi reveals the role of mutation towards drug resistance. The drug intercalates into the DNA and is stabilized through stacking interactions with nitrogenous base. Moreover, it forms hydrogen bonded interaction with Ser83 of GyrA protein. MD simulation of docked complex also corroborates the role of hydrogen bond between quinolone and DNA Gyrase. Hence, the altered geometry at binding pocket and the reorientation of Ser83 affect the binding of the drug which in the modified environment of QBP is no longer capable of forming crucial hydrogen bonded interactions with the mutated residues. The difference in calculated interaction energy between wild type and mutant complex clearly indicates a drop in binding affinity of fluoroquinolones for mutant in comparison to wild type. Thus, these substitutions in the mutant proteins decrease the binding capability and stability of drugs for *st*DNA-Gyrase. The decreased affinity of the drug can be correlated with the increase in MICs in resistant clinical isolates and suggest the probable mechanism for the induced drug resistance in *S*. Typhi.

## Supporting Information

S1 Fig *S*. Typhi GyrA and GyrB. Cartoon representation of model structure (green) of (a) breakage-reunion domain of *st*GyrA and (b) Toprim domain of *st*GyrB of *S*. Typhi superimposed on respective templates (yellow).
(TIF)

S2 Fig Sequence alignment of DNA Gyrase. Multiple sequence alignment of N-terminal (breakage-reunion domain) GyrA of representative Gram positive and Gram negative bacteria including *S*. Typhi using ClustalW. The QRDR region of *S*. Typhi is highlighted in yellow, strictly conserved residues in cyan and site of mutation (residues) in red font. The secondary structure has been indicated for *S*. Typhi.
(TIF)

S3 Fig Snapshot of conformers during MD. Snapshot of MD simulation at 0, 500, 1000, 1500 and 2000ps of time interval for the model complex of DNA Gyrase complex.
(TIF)

S4 Fig Validation of Docking Protocol. Docked position of ciprofloxacin (ball and stick, cyan) in wild type DNA Gyrase of *S*. Typhi (surface) superimposed on crystal position of ciprofloxacin (ball and stick, green) in DNA Gyrase of *Staphylococcus aureus*. Ciprofloxacin occupies a comparable position in the quinolone binding pocket and adopts a similar orientation in both the structures. This validates the docking protocol which is subsequently employed for docking with the mutants.
(TIF)

S5 Fig *st*DNA-Gyrase—ciprofloxacin. Docked position of ciprofloxacin (ball and stick, grey coloured by atom) in the QBP of *st*DNA-Gyrase (cartoon) where DNA is drawn in orange, GyrB in yellow, wild type GyrA in green and mutant GyrA in cyan. Side chain of Ser83 and Asp87 are represented in ball and stick in respective colour and hydrogen bonds are indicated as black dotted lines. (a) Wild type (b) Ser83Phe (c) Ser83Tyr (d) Asp87Tyr and (e) Asp87Gly.
(TIF)

S6 Fig *st*DNA-Gyrase—levofloxacin. Docked position of levofloxacin (ball and stick, blue) in modeled complex of *st*DNA-Gyrase. Rest of the rendering and colouring is same as S5 Fig. (a) Wild type (b) Ser83Phe (c) Ser83Tyr (d) Asp87Tyr and (e) Asp87Gly.
(TIF)

S7 Fig *st*DNA-Gyrase—dextrofloxacin. Docked position of dextrofloxacin (ball and stick, purple) in modeled complex of *st*DNA-Gyrase. Rest of the rendering and colouring is same as S5 Fig. (a) Wild type (b) Ser83Phe (c) Ser83Tyr (d) Asp87Tyr and (e) Asp87Gly.
(TIF)

S8 Fig *st*DNA-Gyrase—moxifloxacin. Docked position of moxifloxacin (ball and stick, magenta) in modeled complex of *st*DNA-Gyrase. Rest of the rendering and colouring is same as S5 Fig. (a) Wild type (b) Ser83Phe (c) Ser83Tyr (d) Asp87Tyr and (e) Asp87Gly.
(TIF)

S1 Table Mutations observed in DNA Gyrase of selected pathogenic bacteria. (DOC)

S2 Table Structural statistics of model structures of *st*GyrA. (DOC)
