# Supplementary figures and images for: Structure Based In Silico Analysis of Quinolone Resistance in Clinical Isolates of Salmonella Typhi from India
Source: PLoS One. 2015 May 11;10(5):e0126560. doi: 10.1371/journal.pone.0126560 (PMC4427296; doi:10.1371/journal.pone.0126560)

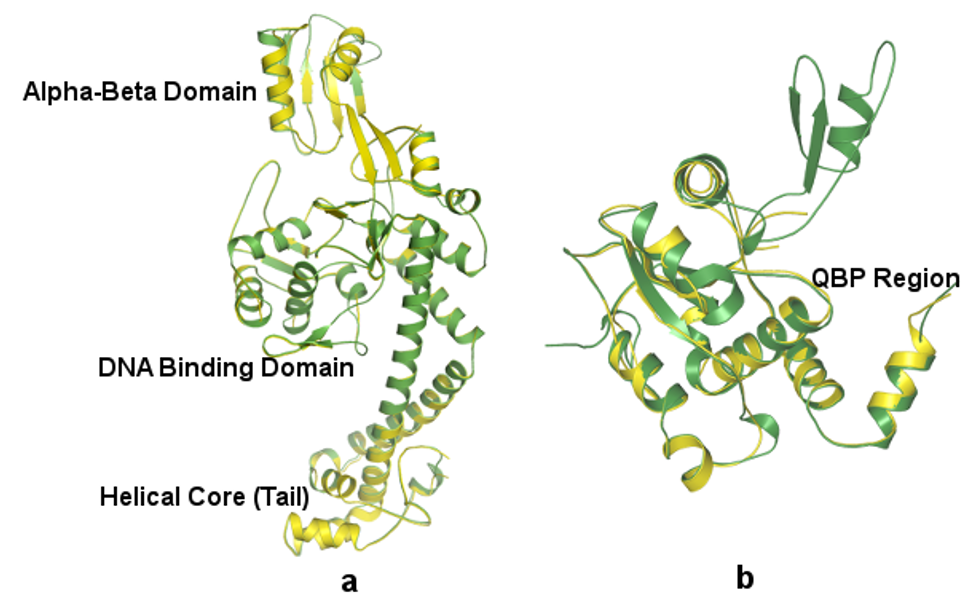

Supplement: S1 Fig — Cartoon representation of model structure (green) of (a) breakage-reunion domain of stGyrA and (b) Toprim domain of stGyrB of S. Typhi superimposed on respective templates (yellow). (TIF) [file pone.0126560.s001.tif]

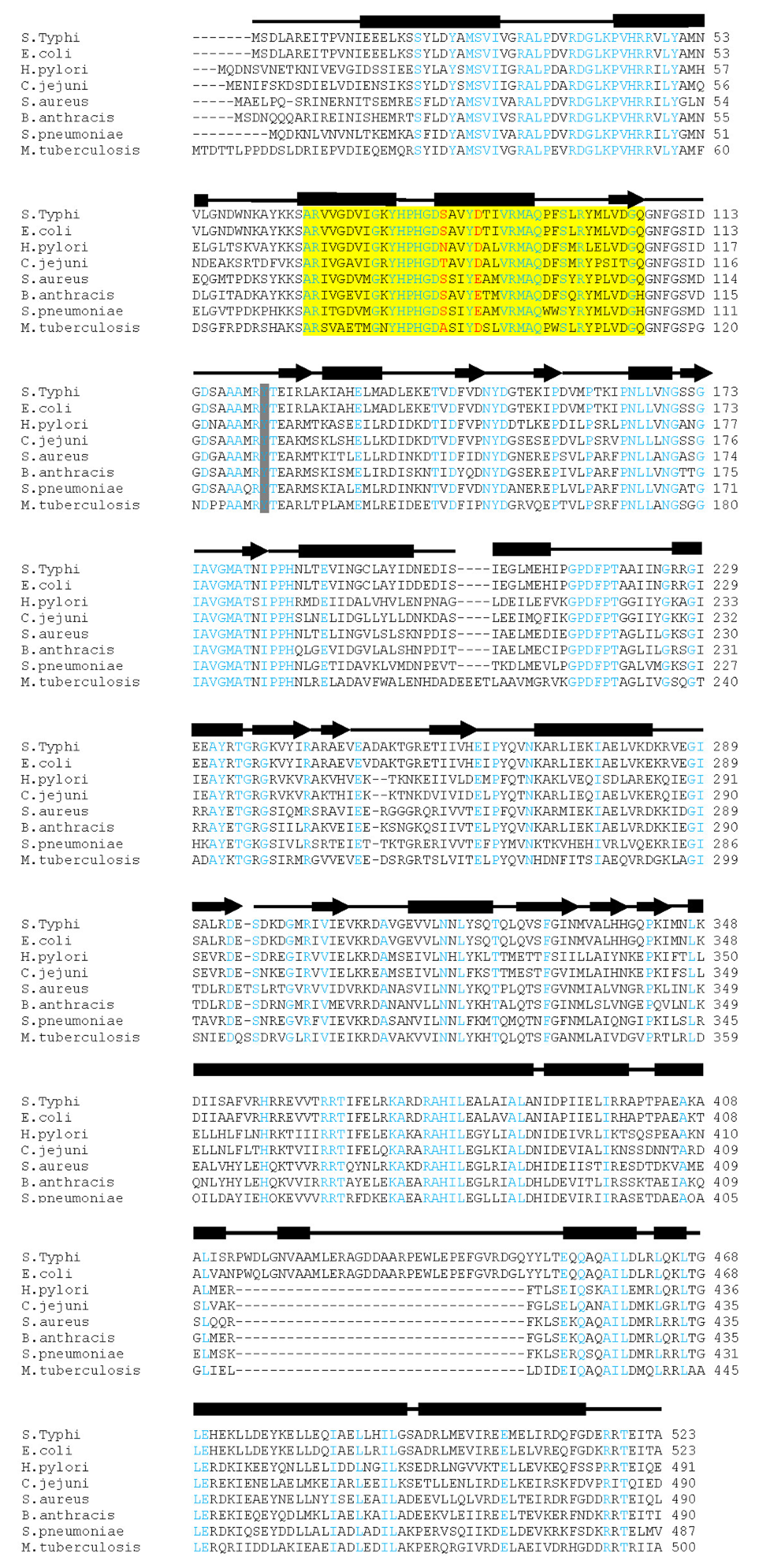

Supplement: S2 Fig — Multiple sequence alignment of N-terminal (breakage-reunion domain) GyrA of representative Gram positive and Gram negative bacteria including S. Typhi using ClustalW. The QRDR region of S. Typhi is highlighted in yellow, strictly conserved residues in cyan and site of mutation (residues) in red font. The secondary structure has been indicated for S. Typhi. (TIF) [file pone.0126560.s002.tif]

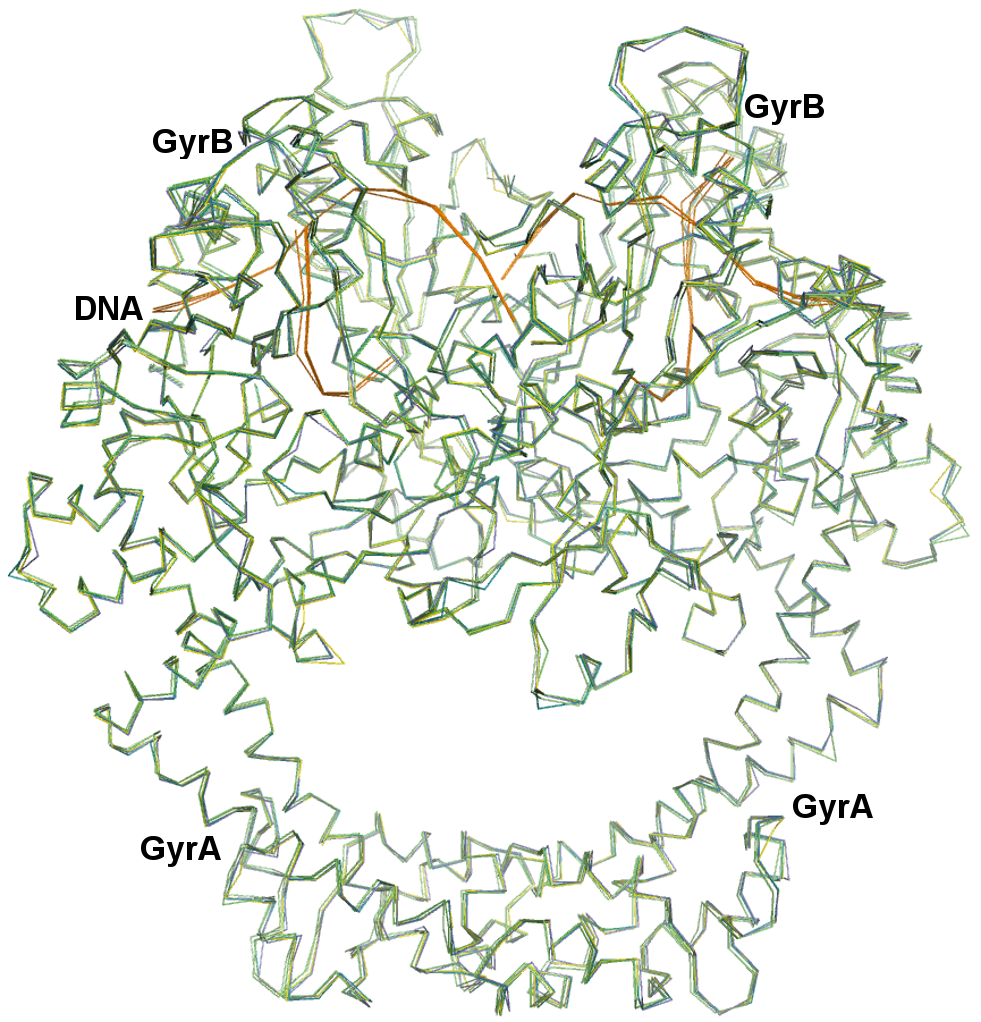

Supplement: S3 Fig — Snapshot of MD simulation at 0, 500, 1000, 1500 and 2000ps of time interval for the model complex of DNA Gyrase complex. (TIF) [file pone.0126560.s003.tif]

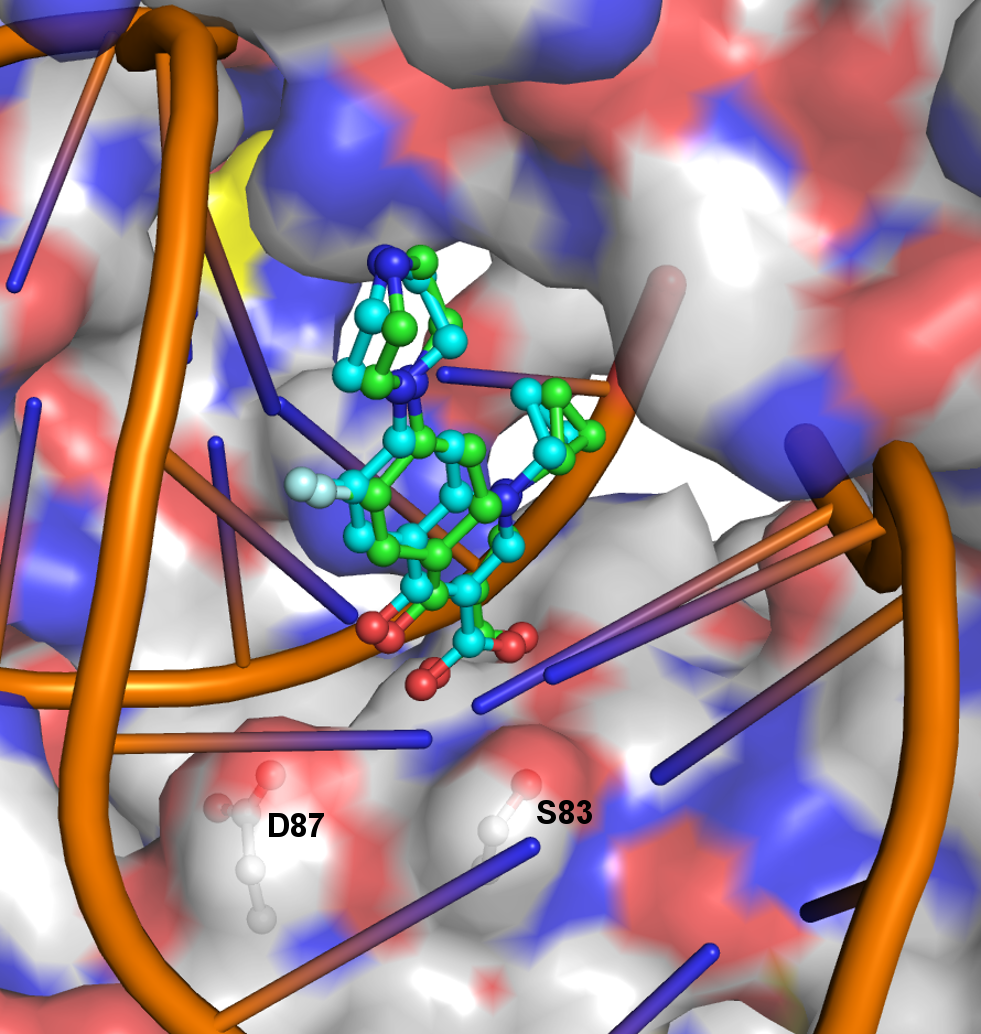

Supplement: S4 Fig — Docked position of ciprofloxacin (ball and stick, cyan) in wild type DNA Gyrase of S. Typhi (surface) superimposed on crystal position of ciprofloxacin (ball and stick, green) in DNA Gyrase of Staphylococcus aureus. Ciprofloxacin occupies a comparable position in the quinolone binding pocket and adopts a similar orientation in both the structures. This validates the docking protocol which is subsequently employed for docking with the mutants. (TIF) [file pone.0126560.s004.tif]

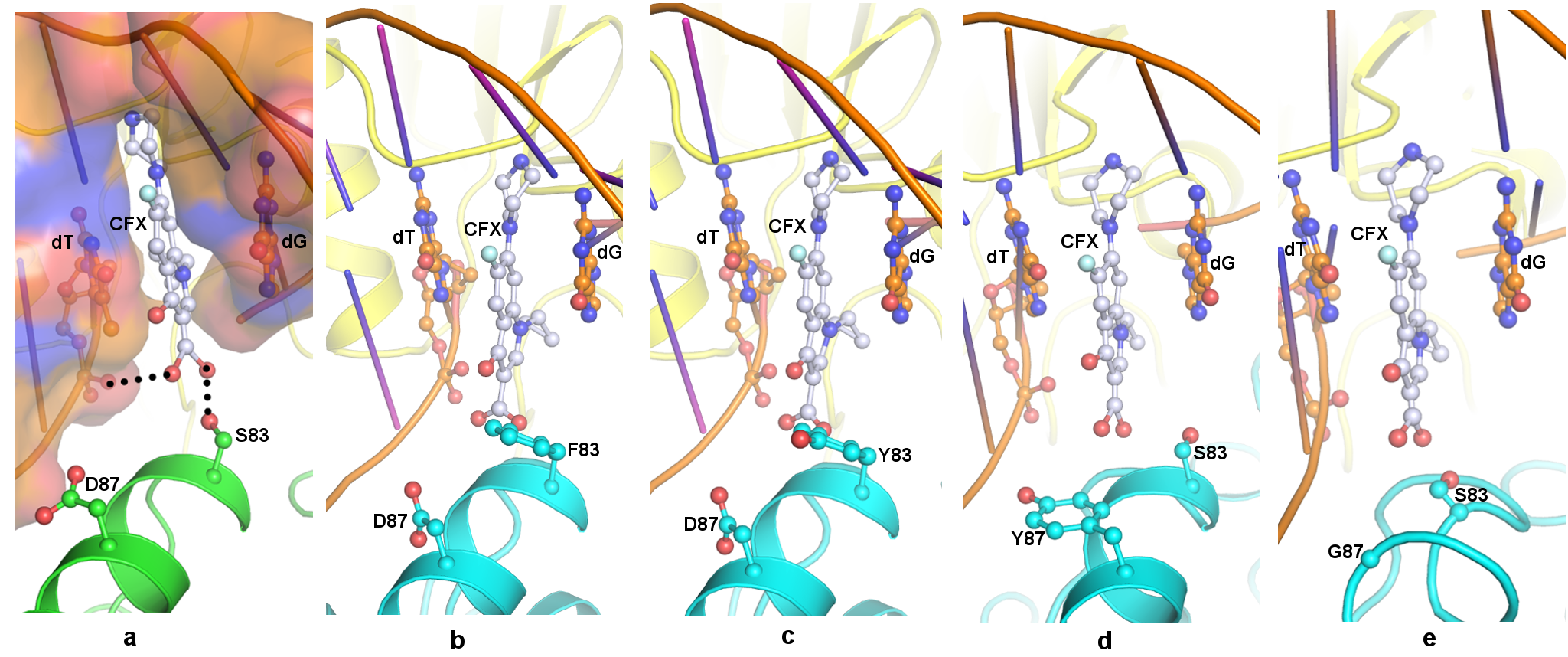

Supplement: S5 Fig — Docked position of ciprofloxacin (ball and stick, grey coloured by atom) in the QBP of stDNA-Gyrase (cartoon) where DNA is drawn in orange, GyrB in yellow, wild type GyrA in green and mutant GyrA in cyan. Side chain of Ser83 and Asp87 are represented in ball and stick in respective colour and hydrogen bonds are indicated as black dotted lines. (a) Wild type (b) Ser83Phe (c) Ser83Tyr (d) Asp87Tyr and (e) Asp87Gly. (TIF) [file pone.0126560.s005.tif]

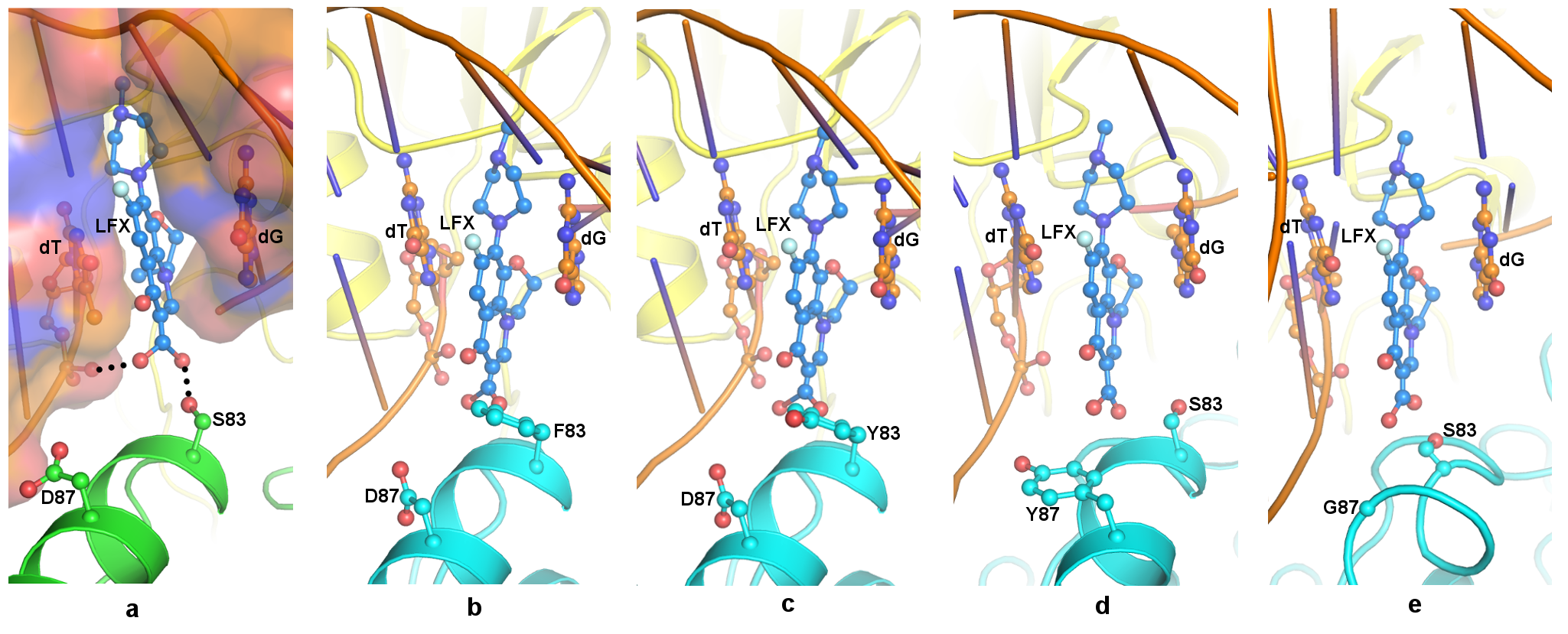

Supplement: S6 Fig — Docked position of levofloxacin (ball and stick, blue) in modeled complex of stDNA-Gyrase. Rest of the rendering and colouring is same as S5 Fig. (a) Wild type (b) Ser83Phe (c) Ser83Tyr (d) Asp87Tyr and (e) Asp87Gly. (TIF) [file pone.0126560.s006.tif]

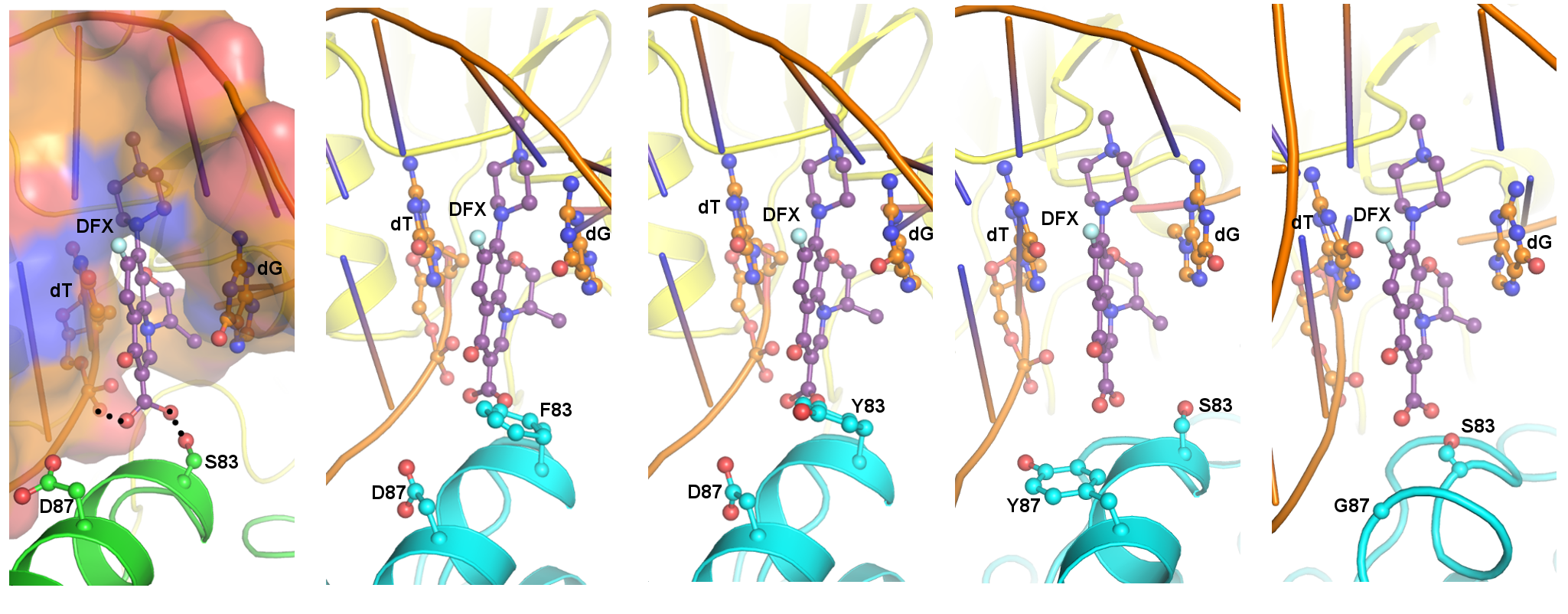

Supplement: S7 Fig — Docked position of dextrofloxacin (ball and stick, purple) in modeled complex of stDNA-Gyrase. Rest of the rendering and colouring is same as S5 Fig. (a) Wild type (b) Ser83Phe (c) Ser83Tyr (d) Asp87Tyr and (e) Asp87Gly. (TIF) [file pone.0126560.s007.tif]

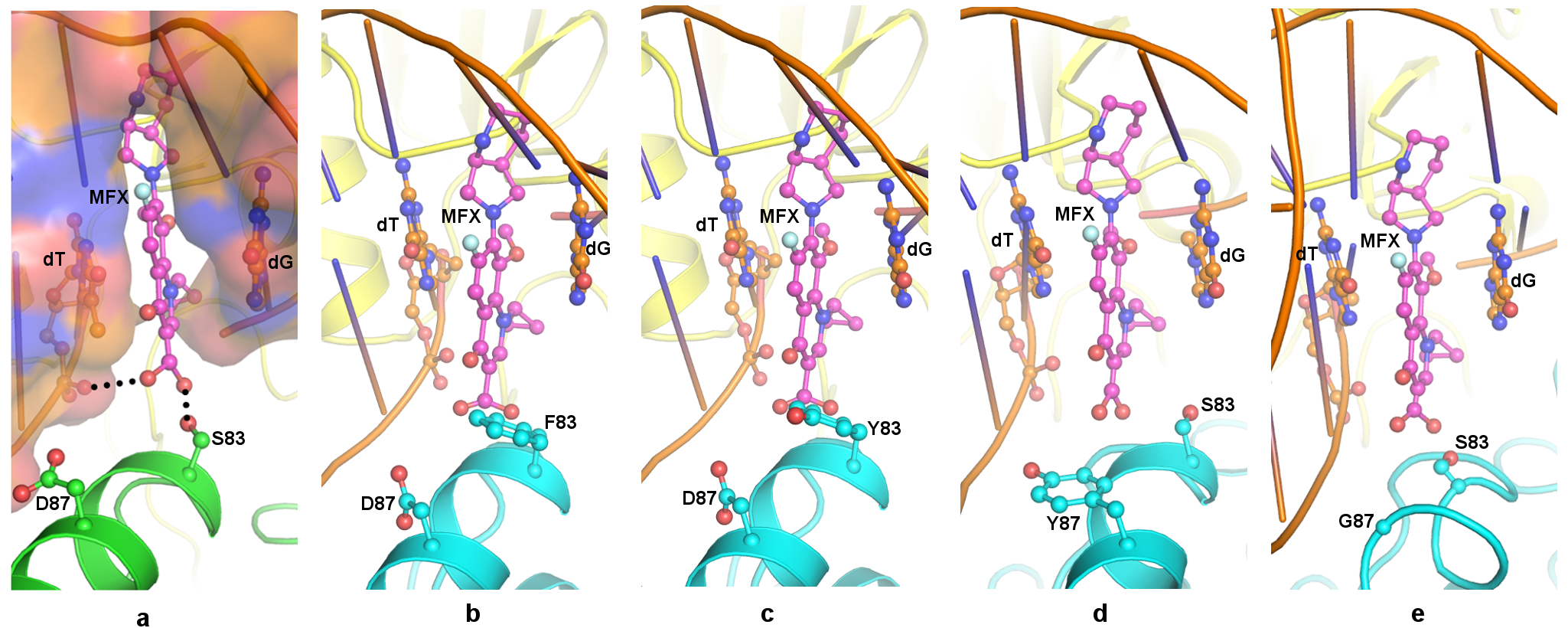

Supplement: S8 Fig — Docked position of moxifloxacin (ball and stick, magenta) in modeled complex of stDNA-Gyrase. Rest of the rendering and colouring is same as S5 Fig. (a) Wild type (b) Ser83Phe (c) Ser83Tyr (d) Asp87Tyr and (e) Asp87Gly. (TIF) [file pone.0126560.s008.tif]
