# Supplementary material for: Structure Based In Silico Analysis of Quinolone Resistance in Clinical Isolates of Salmonella Typhi from India
Source: PLoS One. 2015 May 11;10(5):e0126560. doi: 10.1371/journal.pone.0126560 (PMC4427296; doi:10.1371/journal.pone.0126560)
Supplement: S1 Table — (DOC) [file pone.0126560.s009.doc]

**Table S1: Mutations observed in DNA Gyrase of selected pathogenic bacteria**

| **Bacterial Species** | **Mutations in GyrA**  **(Most commonly observed)** | | **Other mutations** |
| --- | --- | --- | --- |
| *Salmonella* Typhi | Ser83Phe/Tyr **^p,47^**  Ser83Phe+Asp87Asn **^c^**  Ser83Phe+Asp87Gly **^c^** | Asp87Tyr / Gly **^p,47^**  Asp87Asn **^8^** | Leu55Trp,**^a^**  Glu84Lys, **^a^**  Gln106Arg **^a^** |
| *Escherichia coli* | Ser83Leu **^18, 19^** | Asp87Asn/Gly **^18, 19^** | Ala51Val **^b^** |
| *Mycobacterium tuberculosis* | Ala90Val **^20^** | Ala94Gly/His **^20^** | Gly88Ala/Cys **^20^** |
| *Streptococcus pneumoniae* | Ser81Phe/Tyr **^21^** |  |  |
| *Staphylococcus aureus* | Ser84Leu/Lys **^22^** | Asp88Lys/Val **^22^** |  |
| *Acinetobacter baumunnii* | Ser83Leu **^23^** |  | Gly81Val, **^23^**  Ala84Pro **^23^** |
| *Bacillus anthracis* | Ser85Leu **^24^** | Glu89Arg/Gly/Lys **^24^** |  |
| *Helcobacter pylori* | Asn87Lys **^25^** | Asp91Tyr/Asn **^25^** | Val77Ala,**^25^** Ala97Val **^25^** |

p present mutation

**References**

1. Dimitrov T, Dashti AA, Albaksami O, Udo EE, Jadon MM, Albert MJ (2009) Ciprofloxacin-Resistant *Salmonella enterica* Serover Typhi from Kuwait with Novel Mutations in *gyrA* and *parC* Genes. J Clin Microbiol 47: 208-211.
2. Friedman SM, Lu T, Drlica K (2001) Mutation in the DNA Gyrase A Gene of *Escherichia coli* That Expands the Quinolone Resistance-Determining Region. Antimicrob Agents Chemother 45: 2378-2380.
3. Gaind R, Paglietti B, Murgia M, Dawar R, Uzzau, S, Cappuccinelli P, Deb M, Aggarwal P, Rubino S (2006) Molecular characgterization of ciprofloxacin-resistant *Salmonella enterica* serover Typhi and Paratyphi A causing enteric fever in India. J Antimicrob Chemother 58: 1139-1144.
