# Supplementary material for: Structure Based In Silico Analysis of Quinolone Resistance in Clinical Isolates of Salmonella Typhi from India
Source: PLoS One. 2015 May 11;10(5):e0126560. doi: 10.1371/journal.pone.0126560 (PMC4427296; doi:10.1371/journal.pone.0126560)
Supplement: S2 Table — (DOC) [file pone.0126560.s010.doc]

|  | **Wild Type** | **Ser83Phe** | **Ser83Tyr** | **Asp87Tyr** | **Asp87Gly** |
| --- | --- | --- | --- | --- | --- |
| Most favoured regions | 90.8% | 90.8% | 90.8% | 90.8% | 89.9% |
| Additional allowed regions | 8.3% | 8.3% | 8.3% | 8.3% | 9.2% |
| Generously allowed regions | 0.7% | 0.7% | 0.7% | 0.7% | 0.7% |
| Disallowed regions | 0.2% | 0.2% | 0.2% | 0.2% | 0.2% |
| Phi-psi distribution | 0.09 | 0.09 | 0.09 | 0.09 | 0.05 |
| Chi1-chi2 distribution | -0.25 | -0.25 | -0.25 | -0.26 | -0.24 |
| Chi1 only | -0.11 | -0.22 | -0.22 | -0.15 | -0.09 |
| Chi3 & chi4 | 0.36 | 0.30 | 0.30 | 0.35 | 0.36 |
| Omega | -0.09 | -0.09 | -0.09 | -0.08 | -0.09 |
| Main-chain bond lengths | -0.04 | -0.05 | -0.05 | -0.04 | -0.05 |
| Main-chain bond angles | -0.20 | -0.22 | -0.22 | -0.21 | -0.24 |
| Overall G factor | -0.05 | -0.06 | -0.06 | -0.05 | -0.07 |

**Table S2: Structural statistics of model structures of *st*GyrA**
